# Graphene oxide–carbon nanotube hybrid assemblies: cooperatively strengthened OH···O=C hydrogen bonds and the removal of chemisorbed water†
†Electronic supplementary information (ESI) available. See DOI: 10.1039/c7sc00223h



**DOI:** 10.1039/c7sc00223h

**Published:** 2017-05-04

**Authors:** J. D. Núñez, A. M. Benito, S. Rouzière, P. Launois, R. Arenal, P. M. Ajayan, W. K. Maser

**Affiliations:** a Instituto de Carboquímica (ICB-CSIC) , E-50018 Zaragoza , Spain . Email: wmaser@icb.csic.es ; Tel: +34 976 73 39 77; b Laboratoire de Physique des Solides , CNRS , Univ. Paris-Sud , Univ. Paris Saclay , F-91405 Orsay Cedex , France; c Laboratorio de Microscopias Avanzadas , Instituto de Nanociencias de Aragón , Univ. Zaragoza , E-50018 Zaragoza , Spain; d ARAID Foundation , E-50018 Zaragoza , Spain; e Department of Materials Science and NanoEngineering , Rice University , Houston , Texas 77005 , USA

## Abstract

Owing to their great significance for energy storage and sensing applications, multi-layer papers consisting of graphene oxide–carbon nanotube (GO–CNT) hybrid sheets were prepared by *in situ* exfoliation of graphite oxide in the presence of oxidized CNTs (oCNTs). For the first time we elucidate the influence of oCNTs on chemisorbed water (CW), *i.e.* the water molecules inherently bound to the oxygen functional groups (OFGs) of graphene oxide (GO) and responsible for irreversible structural damage upon thermal reduction processes. We show that oCNTs self-assemble onto GO sheets during the liquid phase processing steps by forming cooperatively strengthened OH···O=C hydrogen bonds between the carboxylic groups of the oCNTs and OFGs of GO. At oCNT amounts of about 10 to 15 wt% this leads to the displacement of considerable amounts of CW without altering the original chemical composition of GO. The thermally reduced GO–CNT (rGO–CNT) papers reveal improved sp^2^ character and an enhancement of the specific capacitance by 75% with respect to thermally reduced GO (rGO), largely due to the effective removal of CW by oxidized CNTs. These findings disclose the relevance of the cooperative hydrogen bonding phenomena in graphene oxide paper/film electrodes for the development of improved electrochemical energy storage and sensing devices.

## Abstract 2

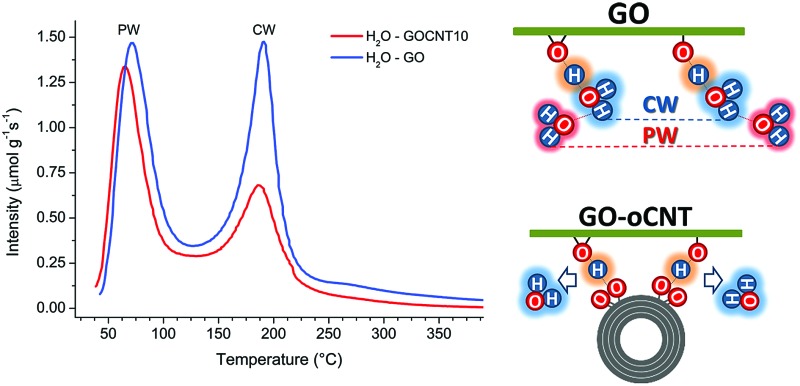
The effective removal of chemisorbed water in graphene oxide by oxidized carbon nanotubes *via* cooperatively strengthened OH···O=C hydrogen bonds.

## Introduction

Multi-layered graphene oxide (GO) assemblies such as thin films or freestanding membranes, likewise called GO papers, have recently attracted great attention for numerous applications in the fields of nanofiltration,^1^ molecular sieving,^2–5^ actuators,^6–8^ sensors^9^ and energy storage.^10–14^ The unique adaptive physicochemical properties of GO sheets control the layered stacking arrangement as well as the conducting properties and, when coupled to facile macroscopic processing from aqueous solutions by simple coating, spraying or filtration methods, they provide a distinctive advantage for this nanocarbon material. The key is the presence of oxygen functional groups (OFGs) located on GO’s basal plane and edges, essentially of the epoxy/hydroxyl and carbonyl/carboxyl types, respectively.^15–19^ They facilitate the hydration/solvation of the GO sheets and impart hydrophilicity. However, when assembled into a stacked layer structure, water remains trapped between the assembled GO sheets.^7,20^ The hydration state, *i.e.* the amount of intercalated water, defines the interlayer distance between the GO sheets. The ultrafast permeation of gases or water is observed in graphene oxide-based membranes, while other types of liquids are blocked, due to tortuous pathways over hydrophobic non-oxidized surfaces corresponding to a decreased interplane distance of about 0.35 nm.^5^ In the fully hydrated state, distances of about 1 nm are reached allowing for the selective sieving of larger sized molecules.^4^ The hydration state may be reversibly controlled by low temperature thermal treatments (typically up to 100 °C), which form the basis for actuating properties,^7^ humidity sensing devices,^9^ or tailoring the proton conductivity in fuel cell devices,^14^ among others.

High temperature thermal treatments are required to convert the electrically non-conducting GO papers into conductive electrode materials.^21–24^ This is based on the removal of the OFGs and the partial restoration of pathways with sp^2^ character.^25^ However, thermal treatments also lead to the removal of carbon atoms from the GO plane, introducing structural vacancies throughout the sheet.^21^ It has been shown that the intercalated water in close proximity to the OFGs facilitates the creation of larger holes, as indicated by enhanced carbonyl formation and the release of CO_2_.^20^ These exothermic water-induced reactions, typically occurring at temperatures beyond 120 °C, are responsible for irreversible damage and even affect the mechanical integrity of the whole macroscopic GO assembly.^21,24^ Water in close contact to the surface of GO (*i.e.* in close proximity to the OFGs) is frequently referred to as physisorbed water.^20,26^ However, the term physisorbed water itself remains somewhat ambiguous. It may be used equally well to describe the part of intercalated water that can be easily and reversibly removed by low-temperature treatments. In this work we will apply the following definitions for the various types of water removed at different stages of the thermal treatment processes: (i) intercalated water: this term will be used generically for all types of water molecules trapped/confined in between the stacked sheets of graphene oxide; (ii) physisorbed water (PW): this refers to the intercalated water, which is not interacting with the OFGs of graphene oxide (at the basal plane or edges). It may cover the terms bulk water, free water, and low dimensional confined water not restricted to translational motion.^27^ This type of water can be reversibly removed by vacuum and/or low temperature treatments.^7,9,14^ (iii) Chemisorbed water (CW): this describes the water molecules that are linked to the OFGs by hydrogen bonds. This type of water is organized in local water clusters, as shown by a very recent molecular dynamics study,^28^ and allows only for localized motions.^27,29,30^ The removal of chemisorbed water occurs at higher temperatures (beyond 100 °C) and implies the decomposition of the OFG groups involved in the hydrogen bonding, and the irreversible damage of GO.^20^ During the course of this work we will show that this terminology is beneficial for better understanding of how to mitigate the harmful deterioration effects caused by the presence of water. This is of special relevance when applying thermal reduction treatments in order to develop rGO electrode materials.

Another strategy to develop conductive GO based electrodes in the form of papers (films), avoiding the need for thermal treatments, is the use of carbon nanotubes and the fabrication of GO–CNT hybrid assemblies.^31–33^ Here, CNTs provide the conductive filler network and additionally contribute to an enhanced pore size distribution. For this reason GO–CNT membranes show great promise in electrochemical applications.^13,31,34–36^ While achieving good performance, to the best of our knowledge, there are no studies on GO–CNT assemblies that address the influence of carbon nanotubes on the critical issue of chemisorbed water.

The aim of this work is to uncover the effects of carbon nanotubes on chemisorbed water in graphene oxide papers. To this end, the GO–CNT hybrid assemblies were prepared by the *in situ* exfoliation of graphite oxide in the presence of different amounts of oxidized carbon nanotubes (oCNTs), and subsequently processed into corresponding multi-layer papers. By using thermogravimetric analysis (TGA), temperature-programmed desorption mass spectroscopy (TPD-MS) and X-ray photoelectron spectroscopy (XPS), we demonstrate that chemisorbed water can be significantly removed by oCNTs assembled onto GO sheets in the liquid phase processing steps. Information on the mechanism for the removal of CW is obtained by Fourier transform infrared spectroscopy (FTIR). The results suggest the formation of a pair of cooperatively strengthened OH···O=C hydrogen bonds between the carboxylic groups of the oCNTs and OFGs on GO sheets, which consequently leads to the displacement of the chemisorbed water. We show that this is best achieved for oCNT concentrations of about 10–15 wt%. Furthermore, improved sp^2^ character for rGO–CNT papers is found, and the preliminary electrochemical studies show significant improvements of the specific capacitance for the rGO–CNT papers with respect to rGO. All these findings can be consistently explained by the effective and non-destructive removal of larger amounts of chemisorbed water due to the presence of a relatively low amount of oCNTs. This results in an enhanced number of freely available OFGs as well as reduced structural damage of GO upon thermal treatment.

## Results and discussion

### Self-assembled GO–CNT hybrid sheets

**Fig. 1:**
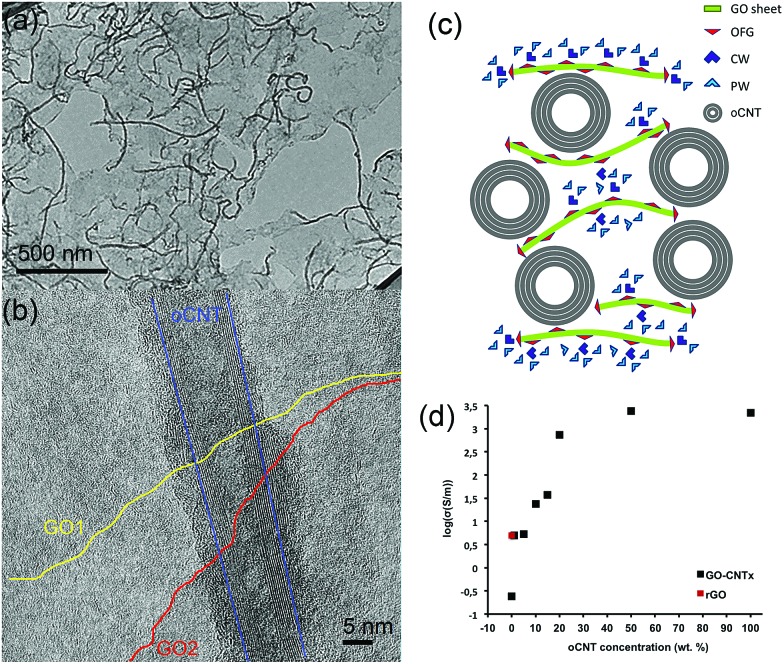
(a) A low-magnification TEM image of the GO–CNT10 sheets showing the self-assembled oCNTs connecting the GO sheets. (b) A high resolution TEM image of a multi-walled oCNT embedded between two GO flakes. Its outer layer is covered by amorphous carbon, which is a characteristic feature for the oCNTs employed. For the sake of clarity, the borderlines of the two observed GO flakes are highlighted in yellow and red. The exterior graphitic layers of the oCNT are marked by a blue line. (c) A scheme illustrating the cross-section of the GO–CNT sheets processed into papers. The green lines with red triangles represent the GO basal plane with the attached oxygen functional groups. The light and dark blue triangles denote the physisorbed (PW) and chemisorbed (CW) molecules, respectively. The oCNTs are depicted as concentric circles. (d) The electrical conductivity of the GO–CNT papers (logarithmic scale) as a function of the oCNT concentration (black squares). The threshold for the change in conductivity towards the oCNT values is located at oCNT concentrations between 10 to 15 wt%. The value for rGO (GO thermally treated at 220 °C) is indicated as a red square.

The excellent water dispersibility of oxidized carbon nanotubes (oCNTs) renders a golden opportunity for effectively combining graphene oxide (GO) with oCNTs. We thus carried out, for the first time to the best of our knowledge, an *in situ* exfoliation process of graphite oxide in the presence of oCNTs (see S1†). In this process, the exfoliated single layer GO sheets act as surfactants^37^ for the oCNTs facilitating the formation of GO–CNT hybrid sheets, which are highly dispersible in water. The TEM images (Fig. 1) show that during this process the oCNTs self-assemble on the GO sheets. Different percentages of oCNTs were used resulting in corresponding hybrids labelled GO–CNT*x* (*x* = 1, 5, 10, 15, 20, 50 wt% of oCNTs). Up to a concentration of 15 wt% of oCNTs, a rather homogeneous coverage of the GO sheets by the oCNTs is achieved (Fig. 1a). The oCNTs are embedded between the GO sheets and interconnect various flakes (Fig. 1b, and S2†). For high loadings, the agglomeration effects of the oCNTs on the GO sheets become dominant (Fig. S2†) owing to the intrinsic dispersion limitations of the oCNTs at concentrations higher than 15 wt%. The well-dispersed GO–CNT sheets are easily processed by flow-directed vacuum filtration into flexible GO–CNT papers (Fig. S3a†). The SEM images (Fig. S3c and d†) reveal an average paper thicknesses of about 30 μm composed by multi-layer stacks of the individual GO–CNT units. The surface and cross-section SEM images (Fig. S3b and d†) both show that the oCNTs are homogeneously distributed throughout the sample. A scheme for the cross-section of the stacked multi-layer paper assembly is presented in Fig. 1c.

The four-point electrical transport measurements as a function of the oCNT concentration (Fig. 1d, Section S4†) reveal a significant increase of the conductivity by 4 orders of magnitude. While GO shows a value of 0.24 S m^–1^, indicative of a mixed ionic–electronic transport mechanism,^14^ the addition of oCNTs provides enhanced electronic conductivity and determines the transport behaviour. An electronic percolation-like behaviour is observed with a threshold at about 10–15 wt% reaching conductivity values of a few hundred S m^–1^. From 20 wt% on, the conductivity approaches 2220 S m^–1^, *i.e.* the value of a pure oCNT paper. Due to being formed by a highly entangled network of oCNTs, this may underline that, beyond a concentration of 20 wt%, the agglomeration effects most likely influence the structural and physical properties of the GO–CNT papers. On the other hand, it is remarkable that below the percolation threshold, at very low oCNT concentrations, the conductivity values exceed the ones obtained for the GO papers thermally treated at 220 °C (rGO). This points out that the additional mechanism may contribute to achieving enhanced conductivities. To shine more light on these issues is the subject of the subsequent systematic studies.

### The removal of chemisorbed water

Two dimensional X-ray diffraction studies (see S5†) carried out for GO papers under vacuum reveal a diffuse isotropic scattering signal, which may be attributed to the presence of chemisorbed water (CW) and oxygen functional groups (OFGs). In the rGO papers (GO papers heated at 220 °C) this signal disappears thus clearly indicating the loss of CW and OFGs upon thermal treatment. The observed shift of the 001 peak from 6.9 Å (GO) to 3.4 Å (rGO) further suggests the removal of oxygen functional groups. For the GO–CNT papers, the diffuse scattering signal seems to be reduced with respect to the intensity of the 10 peaks characteristic of the in-plane structure of graphene and the nanotube walls, which may denote a lower amount of CW. This point is examined in more detail in the following.

**Fig. 2:**
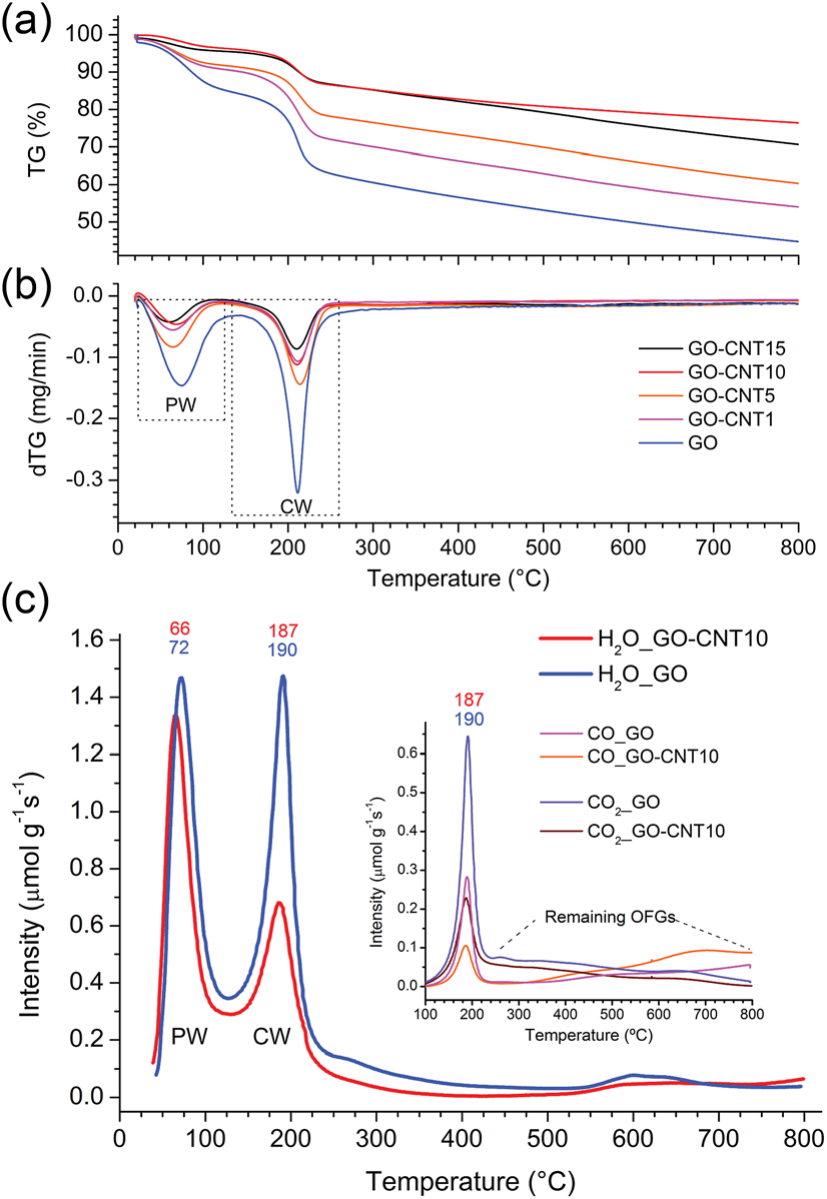
(a) The TGA curves showing the relative thermogravimetric weight-losses for the GO–CNT papers with different concentrations of oCNTs. (b) The derivative thermogravimetric (dTG) curves depicting the temperature zones of maximum weight-losses. The zones associated with the removal of the physisorbed water (PW) and chemisorbed water (CW) are marked by dashed lines. (c) The temperature-programmed desorption mass spectroscopy (TPD-MS) presenting the desorption profiles of H_2_O for GO and GO–CNT10. The first and second peak indicate the removal of the PW and CW, respectively. Inset: the desorption profiles for CO_2_ and CO. Their maxima coincide with the removal of the CW.

Thermogravimetric analyses (Fig. 2a and b) are carried out for the GO–CNT papers with different oCNT amounts. Two important weight losses are observed in the region from 30 °C to 130 °C and between 130 °C to 260 °C. The first one is usually assigned to the desorption of water intercalated between the GO sheets, while the second weight loss is commonly attributed to the decomposition of the oxygen functional groups (OFGs) into CO, CO_2_ and steam.^38^ For GO, weight losses of 15 wt% and 22.5 wt% are encountered for the two regions, respectively. In the case of the GO–CNT samples, the weight losses reveal a systematic decrease from 9.5 wt% (GO–CNT1) to 4 wt% (GO–CNT10) for the first region, and from 19 wt% (GO–CNT1) to 10 wt% (GO–CNT10) for the second region. This trend seems to stabilize for GO–CNT10 with weight loss values similar to those obtained for GO–CNT15. For the GO–CNT samples with higher oCNT concentrations, the TGA behaviour is dominated by the agglomerated oCNTs (more details about the TGA analyses are provided in S6†). Apart from this oCNT agglomeration threshold effect, the observation that GO suffers a weight loss of 22.5 wt% in the temperature range of 130 to 260 °C is rather surprising, if only attributed to the removal of OFGs. Moreover, systematically decreasing the weight loss by increasing the content of oCNTs underlines that more complex processes need to be considered to explain this behaviour.

More information is obtained from a temperature-programmed desorption/mass spectroscopy (TPD-MS) study. Fig. 2c shows the desorption profiles of H_2_O, CO_2_, and CO for the GO and GO–CNT10 papers. Three different temperature regions, comparable to those observed by the TGA, are encountered: (i) from 50 °C to 120 °C, the appearance of a pronounced water desorption peak is observed indicating the removal of water intercalated between the assembled GO sheets, *i.e.* the physisorbed water as defined in the introduction; (ii) from 120 °C to 230 °C the appearance of a second pronounced water desorption peak is accompanied by the desorption of CO_2_ and CO. This is a characteristic feature for the thermal decomposition of GO, *i.e.* the removal of the OFGs (*e.g.* COH, C–O–C) and carbon atoms from its basal plane. While this process induces the formation of larger structural defects (etch holes), it simultaneously allows for the sudden, and actually quite violent, release of larger amounts of CW in the form of steam: GO + H_2_O_CW_ → GO′ + CO + CO_2_ + H_2_ + H_2_O_CW′_. This reaction is initiated by the CW. However, the formation of water itself due to the removal of OFGs is thermodynamically not favorable at 220 °C.^39^ Therefore, the release of larger amounts of water corresponds to not yet reacted clusters of CW molecules suddenly freed through the formed etch holes. Indeed, the existence of larger clusters of CW was confirmed in a very recent molecular dynamics study.^28^ Therefore, the intensity of this desorption peak is a direct measure for the amount of CW attached to GO’s OFGs; (iii) from 230 to 800 °C a rather smooth increase in the desorption intensities for CO and simultaneous decrease in H_2_O and CO_2_ indicates a rather steady decomposition of the more stable OFGs (*e.g.* C=O) remaining on GO.

Comparing GO–CNT10 with GO, no essential differences are encountered for the first region showing a water desorption peak at about 70 °C with almost the same desorption intensity, *i.e.* 1.34 μmol g^–1^ s^–1^ *vs.* 1.47 μmol g^–1^ s^–1^, respectively. However, for GO–CNT10, the desorption intensity of the second water peak at about 190 °C is significantly decreased by almost a factor of 2 with respect to the intensity of the first water peak, reaching now a value of 0.68 μmol g^–1^ s^–1^. The desorption intensities for CO_2_ and CO decrease correspondingly. However, the fact that GO–CNT and GO reveal a similar CO_2_/CO ratio of about 2.2 points out that both the samples suffer analogous decomposition processes. On the other hand, the significantly lower desorption intensity for the second water peak in GO–CNT10 underlines that the presence of oCNTs can considerably lower the amount of CW. This in turn may imply that a smaller amount of OFGs is available to interact with the water molecules. Then again, maintaining the maximum desorption temperature with respect to GO would indicate that the type and distribution of the OFGs are essentially maintained in the GO–CNT samples. Reducing the content of chemisorbed water by only incorporating low amounts of oCNTs, without the need for altering the chemistry of GO, indeed would constitute an outstanding result. This may suggest that oCNTs establish favourable interactions with the OFGs of GO and thus effectively block the attachment of the water molecules yielding reduced amounts of chemisorbed water in GO–CNT. Therefore, XPS and FTIR studies are carried out to gain more information on the chemical composition, removal of chemisorbed water and interactions involved.

**Fig. 3:**
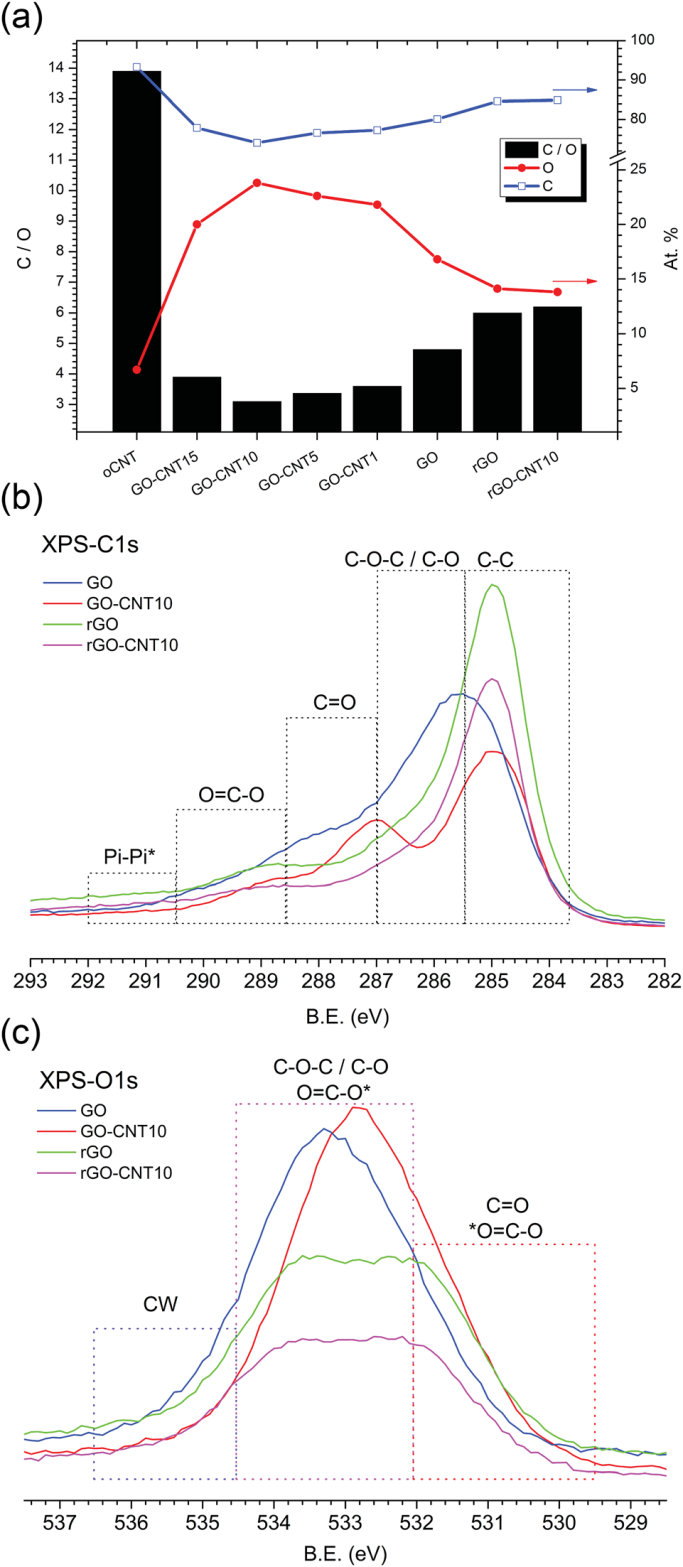
XPS results. (a) The chemical composition of GO–CNT papers as calculated from the XPS survey spectra. For each type of paper, the C/O ratio (black bars, left axis), oxygen content (red line, right axis), and carbon content (blue line, right axis) are presented. (b) The C1s core shell spectra for GO, GO–CNT10, rGO, and rGO–CNT10. (c) The O1s core shell spectra for GO, GO–CNT10, rGO and rGO–CNT10. The dashed frames indicate the range of binding energies for the contributions of carbon, specific groups of the oxygen functional groups, and chemisorbed water (CW).

Fig. 3a shows the chemical composition for GO–CNT papers with different amounts of CNTs as calculated from the XPS survey spectra. With a value of 13.9, oCNT shows the highest C : O ratio of all samples, while GO reveals a C : O ratio of 4.8. The incorporation of oCNTs leads to systematically lower C : O values down to 3.1 for GO–CNT10. At first sight this is counterintuitive but this trend actually reflects the improved removal of the remaining graphitic impurities after the centrifugation step, due to enhanced GO–CNT dispersion quality with respect to GO. Nevertheless, beyond a concentration of 10 wt% the effect of the oCNTs prevails and leads to an increase in the C : O ratio. On the other hand, thermal treatment at 220 °C (partial reduction) leads to C : O ratios of 6.0 and 6.2 for rGO and rGO–CNT10, respectively. This reflects an increase of 25% for rGO and even 100% for rGO–CNT10, with respect to the corresponding non-treated parent papers.

The C1s core shell spectra (Fig. 3b) show the regions assigned to the various components obtained after the fitting procedure. The spectrum for GO is characterized by a relatively low sp^2^ C=C contribution and a dominating broad distribution for the C–O and C–O–C oxygen functionalities followed by lower C=O and C=O–C contributions, indicative of GO’s highly defective structure. On the other hand, the spectrum for GO–CNT10 reveals a larger amount of C=C bonds (due to the presence of oCNTs) and the quite well defined contribution of the O=C–O and C=O groups originating from the oCNTs. These are observed at somewhat lower binding energies compared to the corresponding OFGs present in GO while the contribution of the C–O–C and C–O functional groups seems to be decreased. Once reduced to rGO a higher C=C contribution and significantly reduced amount of all types of functional groups is detected. Remarkably, the spectrum for rGO–CNT10 does not essentially differ from the one of rGO, despite differences in the non-treated parent papers (deconvoluted C1s spectra are shown in Fig. S7†).

A corresponding behaviour can be observed in the O1s spectra (Fig. 3c). The spectrum for GO–CNT10 is shifted to lower binding energies and characterized by a larger distribution of C=O and O=C–O groups indicating the presence of oCNTs. The spectra for rGO and rGO–CNT10 are essentially the same and show a significant loss of the C–O–C and C–O groups (most likely from GO’s basal plane) as well as of the O=C–O contribution. Importantly, a decrease of the intensity is observed in the region between 536.5 and 534.5 eV for GO–CNT10 as well as for rGO and rGO–CNT10, with respect to GO (deconvoluted O1s spectra are shown in S7†). The XPS signals in this region are typically assigned to the presence of water molecules.^40^ Bearing in mind that the XPS experiments were performed under ultra-high vacuum leading to the removal of physisorbed water, this region thus could be attributed to the presence of chemisorbed water. This interpretation then would provide a further hint for the efficient removal of chemisorbed water by the presence of oCNTs.

### Interactions between GO and oCNTs

**Fig. 4:**
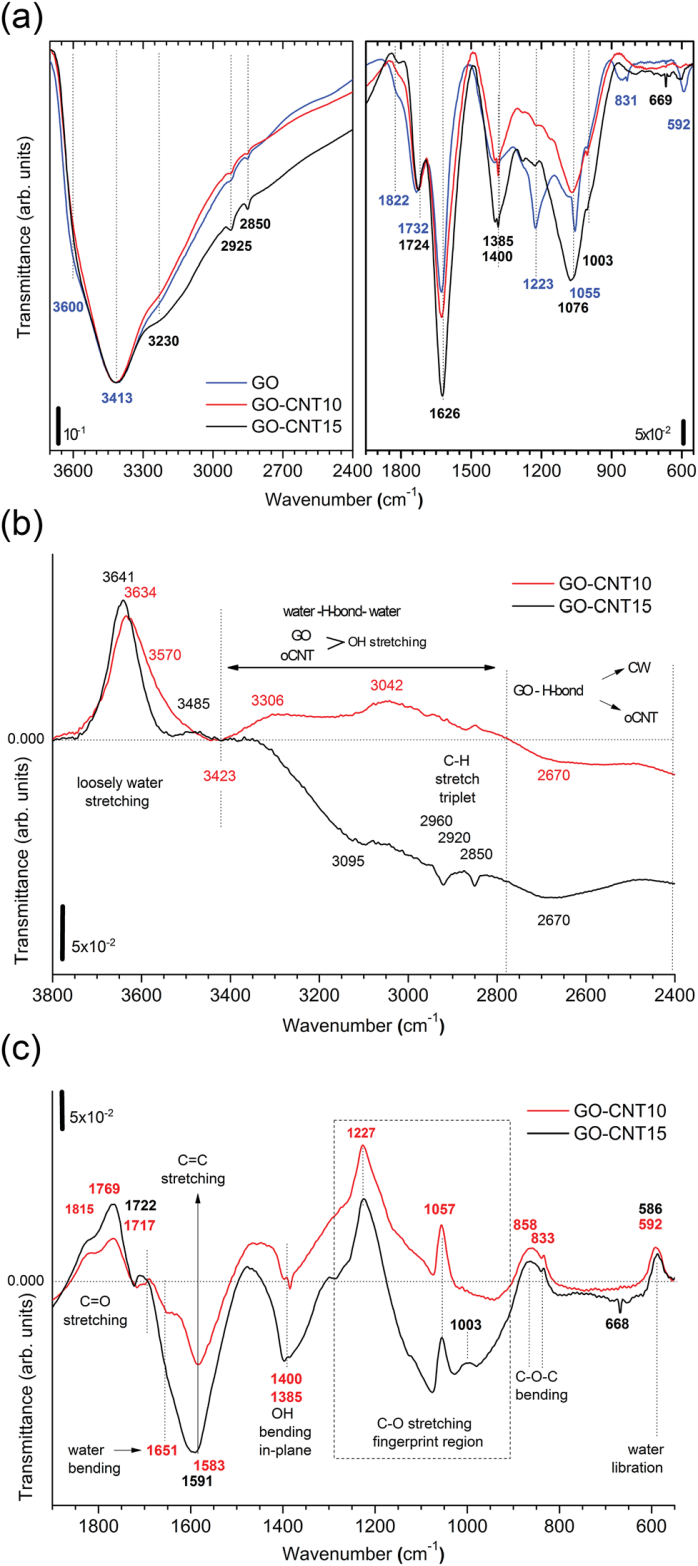
(a) The FTIR spectra for GO, GO–CNT10, and GO–CNT15 presented in transmittance. The left panel shows the broad OH stretch band in the region from 3700–2400 cm^–1^ containing contributions from H_2_O and the functional groups (COOH and C–OH). The right panel depicts the region from 1950–550 cm^–1^ providing information on the vibrational modes for the C=O stretch (1850–1720 cm^–1^), water scissor (1650–1620 cm^–1^), sp^2^ C=C stretch (1500–1600 cm^–1^), OH in-plane bending (1400 cm^–1^ and 1385 cm^–1^), C–O stretch (1223 cm^–1^, 1055 cm^–1^ and 1003 cm^–1^), and C–O–C bending (831 cm^–1^). (b) The transmittance difference spectra obtained after subtracting the spectrum of GO showing the region from 3800 cm^–1^ to 2400 cm^–1^. The positive (upper part) and negative (lower part) values refer to the relative decrease and increase of the GO–CNT signals with respect to GO. A horizontal line marks the zero reference value throughout the wavenumber range. The zones for loosely bound water (3800–3420 cm^–1^), the overlap region for the OH water and oxygen functional groups from GO and oCNTs (3420–2800 cm^–1^), and the cooperatively strengthened OH···O=C hydrogen bonds between GO and oCNTs (2800–2400 cm^–1^) are separated by vertically dashed lines. (c) The transmittance difference spectra showing the region from 1900–550 cm^–1^. The different vibrational modes or respective zones are indicated.

To gain more insight into the removal of chemisorbed water and interactions between GO and oCNTs, detailed FTIR studies of the chemical evolution of GO upon the incorporation of CNTs and thermal reduction are carried out. Fig. 4a shows the infrared transmittance spectra of GO, GO–CNT10 and GO–CNT15. For the sake of discussion, they are divided into two panels. The left panel covers the region from 3700 to 2400 cm^–1^. Down to 3000 cm^–1^ the spectra are characterized by a broad and intense OH-stretching band comprising contributions from H_2_O and the OFGs, such as C–OH, and COOH, in agreement with common tables^41^ and general assignments for GO.^20,42–44^ The weak modes in the region between 3000 to 2800 cm^–1^ are typically assigned to the CH stretching modes, associated with minor hydrocarbon contamination in the spectrometer,^45^ and are not of further interest for this work. More importantly, in contrast to oCNTs (see Fig. S8†), for GO and GO–CNT the OH stretching band extends even further down to wavenumbers as low as 2400 cm^–1^. Although available literature is scarce, such wide broadening has been assigned to the large anharmonic effects involving a pair of cooperatively strengthened OH···O=C intermolecular hydrogen bonds, as shown by theoretical modelling of carboxylic acid dimers, such as formic acid, acetic acid and benzoic acid.^46,47^ Here it was demonstrated that the double-bridged carboxylic acid groups produce complex infrared spectra in which the OH stretch oscillator strength is spread over hundreds of wavenumbers, resulting in a complicated band sub-structure. Given the high number of OFGs in GO, it is reasonable to imagine that OH···O=C hydrogen bonds can form, especially between the OFGs of stacked GO sheets, as suggested by Buchsteiner *et al.* ^27^ The presence of these intermolecular bonds may be significantly enhanced in multilayer papers and thus lead, as described above, to important anharmonic effects responsible for the complex broadening of the OH stretch band.

In fact, Zhang *et al.* ^42^ observed a rather pronounced band between 2800 and 3000 cm^–1^ for a high-temperature reduced GO film deposit and tentatively assigned it to the presence of dimeric carboxylate associated with interactions between the edges of the neighbouring graphene oxide sheets. However, having a closer look, the OH stretching band even reaches down to 2300 cm^–1^ and shows a rather complex band-substructure. While not further commented on in literature, we suggest that the broadly structured band in the region between 3000 cm^–1^ and 2400 cm^–1^ is a characteristic feature of multi-layered GO materials and contains essential information on the intermolecular OH bonding between the OFGs of the stacked GO sheets. Their interaction can be influenced by the presence of oCNTs, as we will demonstrate below.

The right panel depicts the region from 1950 to 550 cm^–1^. The relevant peaks are assigned according to common tables^41^ and in agreement with the literature on GO:^20,42–44^ the C=O stretching modes (1822 cm^–1^ and 1734 cm^–1^), water scissor mode (1626 cm^–1^), OH in-plane bending modes (1400 cm^–1^ and 1385 cm^–1^), C–O stretching modes (1223 cm^–1^, 1055 cm^–1^ and 1003 cm^–1^), C–O–C bending mode (831 cm^–1^), and water libration mode (592 cm^–1^). GO–CNTs exhibit similar spectra with the red-shifted C=O (1724 cm^–1^) as well as the blue-shifted C–O (1076 cm^–1^), and water libration mode (610 cm^–1^). However, C–O (1223 cm^–1^) and C–O–C (831 cm^–1^) cannot be clearly identified anymore. The disappearance of these C–O and C–O–C modes belonging to the out-of plane OFGs of GO suggests that interactions of GO with oCNT may involve these types of OFGs.

A closer analysis is performed using the difference-spectra of GO–CNT10 and GO–CNT15 obtained after subtracting the spectrum of GO (Fig. 4b and c). The positive and negative values indicate the relative decrease and increase of the GO–CNT signals with respect to GO. First we focus on the region involving the OH stretching modes (Fig. 4b). We observe a broad band centred at 3641 cm^–1^ and 3634 cm^–1^ for GO–CNT10 and GO–CNT15, respectively. In agreement with a previous study these peaks are assigned to the loosely bound water, *i.e.* the low coordinated water molecules not interacting with OFGs (free water), probably trapped (physisorbed) within more hydrophobic nanochannels.^48^ Below 3400 cm^–1^, for the oxidized carbon materials, one must consider the OH stretching modes of GO and oCNTs overlapping with the OH stretching modes of the (highly coordinated) physisorbed water molecules. The gain in negative transmittance difference between 3400 cm^–1^ and 2800 cm^–1^ simply indicates the presence of increased amounts of OFGs from oCNTs, as can be seen for GO–CNT15. On the contrary, a positive transmittance difference in this region, regardless of the OFG contribution of oCNTs, is indicative of the (partial) removal of physisorbed water, which clearly can be seen for GO–CNT10.

Importantly, in the region of strongly intermolecular H-bond coordination between 2800 cm^–1^ and 2400 cm^–1^, a broad negative band centred at about 2670 cm^–1^ is observed for both GO–CNT10 and GO–CNT15. This band forms as a result of the broadening effect of the OH stretching modes with respect to GO and can be considered as evidence for the cooperatively strengthened OH···O=C intermolecular hydrogen bonds, as discussed above, between GO and oCNT. Here the OFGs, most likely hydroxyl and epoxy groups of GO and carboxyl groups of oCNTs, act as H-bond acceptors pulling alternatively on the shared H atom towards GO and oCNT. This increases the dipole moment and lowers the frequency of vibration. H-bonding also explains the GO–CW interaction whereby the OFGs and water molecules both act as H-bond acceptors. After the insertion of the oCNTs, H-bonding through O=C–O is favoured and the chemisorbed water is removed from GO. Nevertheless, not all of the OFGs of GO interact with the OFGs of the oCNTs. The extent of the interaction depends on the efficiency of the insertion of the oCNTs in between the GO sheets achieved in the *in situ* exfoliation process, best achieved at oCNT concentrations of about 10–15 wt%.

We now discuss the region below 2000 cm^–1^ (Fig. 4c). Both GO–CNT10 and GO–CNT15 show a similar tendency towards the positive intensity values for the C=O stretching modes (1815 cm^–1^, 1769 cm^–1^ and 1722 cm^–1^) and for the C–O stretching modes (1227 cm^–1^, 1057 cm^–1^ and 833 cm^–1^), indicating a restricted vibration activity with respect to GO. By contrast, other C–O stretching modes become activated between 1057 cm^–1^ and 833 cm^–1^. Although some of these vibrations can be attributed to the C–O modes of the OFGs located on the oCNTs (Fig. S8†), the complete deactivation of the C–O and C–O–C modes at about 1223 cm^–1^ and 831 cm^–1^ provides strong evidence for the formation of H-bonding between GO and oCNT *via* C–O and C–O–C type OFGs located at the basal plane of GO. The vibrational restrictions for the 1223 cm^–1^, 1055 cm^–1^ and 831 cm^–1^ peaks can be affected by H-bonding between GO and CW, as shows the increased intensity for rGO obtained after thermal treatment (Fig. S8†). However, in this case the H-bonding is not strong enough to completely silence these modes, as is the case for H-bridged GO–CNT. In addition, the ability of the GO–CNTs to remove CW is further supported by the strong decrease of the water bending mode at 1651 cm^–1^ and the appearance of the C=C stretching mode at 1591 cm^–1^.

**Fig. 5:**
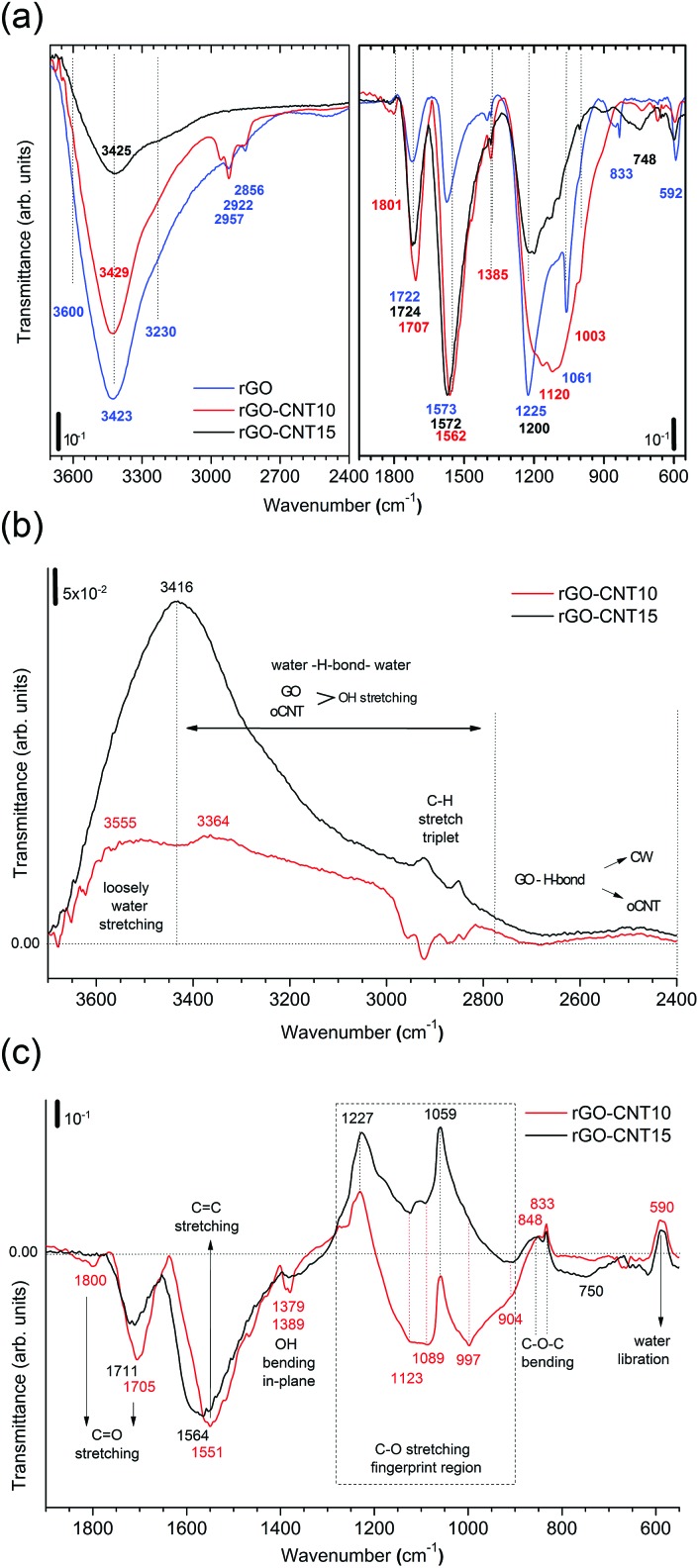
(a) The FTIR spectra for rGO, rGO–CNT10 and rGO–CNT15 presented in transmittance. The left panel shows the broad OH stretch band in the region from 3700–2400 cm^–1^ containing the contributions from H_2_O and functional groups (COOH and C–OH). The right panel depicts the region from 1950–550 cm^–1^ providing information on the vibrational modes for the C=O stretch (1850–1700 cm^–1^), sp^2^ C=C stretch (1500–1600 cm^–1^), OH in-plane bending (1400 cm^–1^ and 1385 cm^–1^), C–O stretch (1230–1200 cm^–1^, 1060–1055 cm^–1^ and 1003 cm^–1^), and C–O–C bending (831 cm^–1^). (b) The transmittance difference spectra obtained after subtracting the spectrum of rGO showing the region from 3800–2400 cm^–1^. The horizontal line marks the zero reference value throughout the wavenumber range. The zones for the loosely bound water (3800–3420 cm^–1^), overlap region for OH water and oxygen functional groups from GO and oCNTs (3420–2800 cm^–1^), and cooperatively strengthened OH···O=C hydrogen bonds between GO and oCNTs (2800–2400 cm^–1^) are separated by vertically dashed lines. (c) The transmittance difference spectra showing the region from 1900–550 cm^–1^. The different vibrational modes or respective zones are indicated.

The infrared spectra of thermally treated samples rGO, rGO–CNT10 and rGO–CNT15 are presented in Fig. 5a and the evolution of the vibrational modes is followed using difference spectra of rGO–CNT10 and rGO–CNT15 obtained after subtracting the spectrum of rGO (Fig. 5b and c). The OH stretching region (Fig. 5b) reveals the effective removal of all different types of water with respect to rGO. In the case of rGO–CNT15, the removal of loosely bound/free water seems to be particularly enhanced. This can be attributed to the ease of diffusion of this type of trapped water throughout the wider pore structure of the hybrid assembly, which is achieved at high oCNT loadings. Importantly no significant changes are observed in the region of 2800 to 2400 cm^–1^ characteristic of the H-bond coordination between GO and the oCNTs. However, showing the positive values over the whole range indicates the permanent de-bonding of H-bridges between rGO and the oCNTs.

The second region (Fig. 5c) reveals that the sp^2^ structure for the rGO–CNT hybrids is preserved. This is even more evident in the case of rGO–CNT10 where the C=C stretching mode is red-shifted to 1551 cm^–1^, which is distinctive of the restoration of the sp^2^ conjugated carbon network. Additionally, rGO–CNT10 shows the highest relative intensity for the C=O and C–O modes, indicative of limited decomposition of the OFGs during thermal treatments with respect to rGO.

**Fig. 6:**
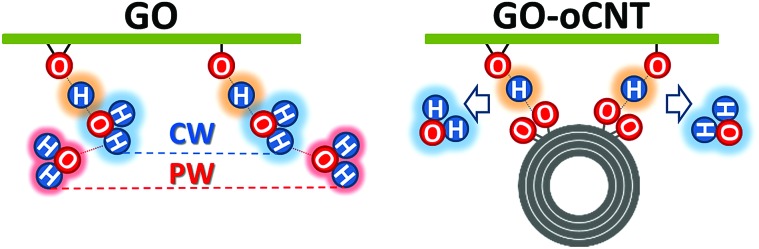
A model for the cooperative hydrogen bonding between the chemisorbed water (CW) and oxygen functional groups (OFGs) on GO (left) and between the carboxylic groups of the oCNTs and the OFGs on GO (right) leading to the removal of chemisorbed water. The basal plane of GO is indicated as a green line to which the OFGs (epoxy and hydroxyl groups) are attached. CW is marked by a blue cloud, physisorbed water (PW) by a red cloud, and cooperatively shared H atoms by an orange cloud. An oxidized multiwall carbon nanotube is represented by concentric circles to which carboxylic groups are attached. The model is equally valid for the stacked sheets of GO and GO–CNTs where the cooperative hydrogen bonding leads to the connection of the neighbour sheets.

Furthermore, our findings reveal that the rGO–CNT hybrids can improve the electrical conductivity by two orders of magnitude with respect to GO while maintaining the original distribution of the OFGs for GO. The key is the formation of cooperative H-bonds between GO and the oCNTs yielding the effective removal of chemisorbed water as a source for irreversible structural damage upon thermal treatments. In agreement with all of the former studies, this is best achieved for oCNT concentrations at around 10–15 wt%, which allow for both a rather homogeneous interaction with GO sheets and a uniform incorporation between the multilayer GO–CNT stacks (please note that at higher concentrations oCNT agglomeration effects prevail). A scheme is shown in Fig. 6 illustrating the interactions between GO and the oCNTs, based on the cooperatively strengthened OH···O=C intermolecular hydrogen bonds established during the liquid phase processing steps and leading to the displacement of CW.

Last but not least, the preliminary electrochemical studies (see S9†) show that the GO–CNT papers serve as conductive electrode materials. The highest values for the specific capacitance of up to 156 F g^–1^ are achieved for oCNT concentrations of 10–15 wt% in the thermally reduced rGO–CNT papers. This corresponds to a significant improvement of 75% with respect to the rGO paper. The results can be consistently explained with the enhanced access of the electrolyte to the active sites of GO facilitated by the oCNTs homogeneously embedded in the multilayer paper at concentrations below or near the agglomeration threshold. Their presence not only ensures enhanced conductivity and increased pore size by avoiding re-stacking of reduced rGO upon thermal treatments as underlined in the literature,^13,34–36^ but equally or even more importantly, provides enhanced access of the electrolyte to the non-damaged GO sheets and to a higher number of remaining oxygen functional groups of GO due to the effective removal of the chemisorbed water. Therefore, the formation of cooperative hydrogen bonds between the oCNTs and GO and the effective displacement of the CW directly impacts on the performance of electrochemical devices.

## Conclusions

For the first time, the influence of oxidized carbon nanotubes on chemisorbed water in graphene oxide is elucidated. It is demonstrated that relatively small amounts of oCNTs can effectively remove the chemisorbed water molecules in GO. This is achieved through the formation of cooperatively strengthened OH···O=C hydrogen bonds between the carboxylic groups of the oCNTs and OFGs on GO when applying an *in situ* liquid phase exfoliation process of graphite oxide in the presence of water dispersible oCNTs. The macroscopic multi-layer paper assemblies prepared from the highly stable GO–oCNT hybrid dispersions serve as an electrode material in electrochemical applications and show superior performance with respect to the specific capacitance. This is a direct consequence of the specific interactions between GO and the oCNTs and the removal of the chemisorbed water providing enhanced sp^2^ character, decreased structural damage upon thermal treatment, and improved access of the electrolyte to the non-damaged GO and remaining OFGs. These findings uncover the general importance of the cooperative OH···O=C hydrogen bonding phenomena in GO and derived macroscopic assemblies as well as its effective manipulation by the oCNTs (or even other types of oxidized dispersible materials) as a tool for the development of improved GO based electrodes in electrochemical energy storage and sensing devices.
