# Supplementary material for: Graphene oxide–carbon nanotube hybrid assemblies: cooperatively strengthened OH···O[double bond, length as m-dash]C hydrogen bonds and the removal of chemisorbed water
Source: Chem Sci. 2017 May 4;8(7):4987–95. doi: 10.1039/c7sc00223h (PMC5625303; doi:10.1039/c7sc00223h)
Supplement: Supplementary file 1 [file SC-008-C7SC00223H-s001.pdf]

Electronic Supplementary Material (ESI) for Chemical Science.
This journal is © The Royal Society of Chemistry 2017

Electronic Supplementary Information (ESI)

Graphene oxide - carbon nanotubes hybrid assemblies: Cooperatively strengthened OH···O=C hydrogen bonds and removal of chemisorbed water

J. D. Núñez[a], A. M. Benito[a], S. Rouzière[b], P. Launois[b], R. Arenal[c,d], P. M. Ajayan[e], and W. K. Maser[a*]

[a] *Instituto de Carboquímica (ICB-CSIC), E-50018 Zaragoza, Spain*

[b] *Laboratoire de Physique des Solides, CNRS, Univ. Paris-Sud, Université Paris Saclay, 91405 Orsay Cedex, France*

[c] *LMA-INA Univ. Zaragoza, E-50018 Zaragoza, Spain Laboratorio de Microscopías Avanzadas, Instituto de Nanociencia de Aragón, Univ. Zaragoza, E-50018 Zaragoza, Spain*

[d] *ARAID Foundation, E-50018 Zaragoza, Spain*

[e] *Department of Materials Science and NanoEngineering, Rice University, Houston, Texas 77005, USA*

* E-mail: wmaser@icb.csic.es; Tel: +34 976 73 39 77

S1. EXPERIMENTAL

1.1 Chemicals

Graphite flakes (flake size > 100 mesh) were purchased from Sigma-Aldrich (Ref 332461); Multi-wall carbon nanotubes (Nanocyl 7000^{TM}, average diameter 9.5 nm, average length 1.5 µm, carbon purity 90%) were acquired from Nanocyl S.A; sodium nitrate, potassium permanganate, hydrazine monohydrate were obtained form Sigma-Aldrich; sulfuric acid (96%), hydrochloric acid (37%), hydrogen peroxide (33%) were purchased from Panreac S.A.

1.2 Preparation of dispersions of graphene oxide

Graphite oxide was prepared using a modified Hummers' method from graphite flakes by oxidation with $NaNO_3$, H_2SO_4 and $KMnO_4$ in an ice bath as reported elsewhere.[1] Briefly, 5 g of graphite flakes and 3.75 g of $NaNO_3$ are oxidized with 170 mL of concentrated solution of H_2SO_4 under vigorous magnetic stirring for 30 min. Afterwards, 25 g of $KMnO_4$ is added in small doses keeping the temperature below 5 ºC meanwhile the dispersion is stirred for 30 minutes. At this point, the dispersion turns green and the ice bath is removed while the dispersion remains stirring at 35 ºC for 2 hours. Subsequently, 250 mL of distilled water and 20 mL of H_2O_2 (33%) are slowly added and the dispersion is stirred for 1 hour. Finally, the obtained brown paste is filtered and washed repeatedly with a solution of distilled water and HCl (10:1). The dispersion of graphene oxide (GO) was obtained by bath ultrasonication of the prepared graphite oxide powder in distilled water (0.3 mg/mL) for 30 min, followed by 30 min of mild pulse ultrasonication (Hielscher UP400S) power amplitude 100 %, cycle 0.5 seconds. Subsequently, centrifugation at 18000g was applied for 60 min resulting in a light brown-coloured solution of exfoliated GO.

1.3 Processing graphene oxide into multilayer paper assemblies

Freestanding flexible GO papers were prepared by vacuum assisted flow filtration of 500 mL of the aqueous GO, with a concentration of 0.15 mg/mL, using an Isopore filter (ø 47 mm, pore size 1.2 µm). The paper assemblies were rinsed with water and dried overnight before being peeled-off from the filter.

1.4 Preparation of oxidized carbon nanotubes

Oxidized multiwall carbon nanotubes (oCNTs) were prepared by refluxing commercial carbon nanotubes in 9.5M HNO_3 for 18 h. After oxidation the resultant material was filtered, repeatedly washed with distilled water until neutral pH was achieved, and dried at 80 ºC under vacuum for 12 h.

1.5 Synthesis of GO-CNT hybrids and processing into multilayer papers

GOCNT samples were prepared by carrying out an *in-situ* exfoliation of graphite oxide, obtained by the modified Hummer method described above, mixed with a desired relative content of oCNTs and labelled as GO-CNTx (x = 1, 5, 10, 15, 20, and 50 wt. %). Afterwards, the processing route for the preparation of GO-CNT papers follows a similar procedure intended for the preparation of GO paper samples. First, the mixture of graphite oxide and oCNTs powders is bath sonicated in distilled water (0.3 mg/mL) for 30 min, followed by 30 min of mild pulse ultrasonication (Hielscher UP400S) power amplitude 100 %, cycle 0.5 seconds. Subsequently, centrifugation at 18000g was applied for 60 min resulting in dark brown-coloured dispersion. Freestanding flexible GO-CNT samples were prepared by vacuum assisted flow filtration of 500 mL of the aqueous GO-oCNT dispersion using an Isopore filter. The papers were rinsed with water and dried overnight before being peeled-off from the filter. For the sake of comparison, a pure oCNT paper was prepared following the same procedure used for GO-CNTs.

1.6 Thermal reduction treatment

The GO and GO-CNT samples were heated at 220 ºC in a preheated forced convection furnace for a time of 30 minutes. The resulting partially reduced samples are labelled rGO and rGO-CNT, respectively.

1.7 Characterization techniques

High-resolution transmission electron microscopy (HRTEM) images were acquired using a high-resolution Cs-corrected FEI-Titan-Cube microscope working at 80 kV. The paper samples were ripped off applying pulsed sonication in ethanol for 45 min and a drop of the obtained suspension was placed onto a copper grid coated with carbon film.

A Hitachi S-3400N Scanning electron microscope (SEM) and a Carl Zeiss MERLIN field-emission scanning electron microscope (FESEM) were employed to characterize GO-CNT samples on the surface and along its cross-section.

Electrical conductivity of the GO-CNT papers samples was measured in a co-lineal four-point probes method[2] using a Keithley 2000 System SourceMeter current source.

X-ray scattering measurements were performed: (i) in air, on a copper rotating anode generator, ($\lambda_{CuK\alpha}$ = 1.542 Å) monochromatised by multilayer W/Si optics. (ii) under vacuum, on a molybdenum rotating anode generator ($\lambda_{MoK\alpha}$ = 0.711 Å) monochromatised by a doubled-curved graphite (002) monochromator. In the first experiment, XRD patterns were recorded on a MAR research X-ray sensitive 345 mm plate detector with 150 µm pixel size placed after the sample at a distance of 100 mm. In the second one, XRD patterns were recorded on an X-ray imaging-plate (IP) with 200µm pixel size, positioned at 100 mm behind the sample; both sample and IP were placed inside a chamber under vacuum to obtain scattering patterns with high signal over background ratio.

Determination of the average layer number and of the interlayer distance for GO and graphitic sheet assemblies are performed by fitting the profile of their respective XRD 001 peaks, using the interference function for diffraction.[3]

$$G(Q) = \sum_{M} \left(\frac{sin\frac{MQd_{001}}{2}}{sin\frac{Qd_{001}}{2}} \right)^2$$

with M being the average layer number, d_{001}, the interlayer distance and Q, the scattering wavevector.

TGA studies were performed in a SETARAM Setsys Evolution apparatus. The paper samples were heated with a constant ramp of 10 ºC/min until 800 ºC under an argon flow of 50 mL/min. Temperature-programmed desorption coupled to a mass spectrometer (TPD-MS) was operated on a Micromeritics equipment Autochem II 2920. The paper samples were heated with a constant ramp of 10 ºC/min until 800 ºC under a flow of 50 ml/min of argon while the concentration of the desorbed molecules were monitored according to their molecular mass (18, 28, 44) for water, carbon monoxide and carbon dioxide respectively.

XPS measurements were carried out on paper samples on an ESCAPlus Omicron spectrometer using a monochromatized Mg X-ray source (1253.6 eV) under ultrahigh vacuum. CasaXPS software was used to calculate the chemical compositions and to perform the deconvolution of the broad bands. For this purpose a Shirley background was subtracted and the spectra were corrected by fixing the position of the C=C peak for all spectra to 285 eV.

FTIR spectra were recorded on a Bruker Vertex 70 instrument in the range of 4000 cm^{-1} to 400 cm^{-1} operating at a resolution of 4 cm^{-1}. The signal was averaged over 32 scans. During the time of acquisition, the sample compartment is continuously purged with a flow of dry air. The papers were ground into powder and mixed with KBr to make a pellet. The sample/KBr weight ratio was

approximately 1/100. Spectra are background corrected and presented in transmittance, normalized to the mode of maximum intensity.

An Autolab PGSTAT302 potentiostat was employed for the electrochemical characterization of a two-electrode electrolytic cell system comprising two freestanding paper-like electrodes separated by a glass fiber membrane soaked in (1M) Na_2SO_4 or (0.5M) H_2SO_4 electrolytes at room temperature. Cyclic voltammetry (CV) was carried out in a potential range of -0.5 to 0.5 V at voltage scan rates of 5, 10, 50, and 100 mV/s. The specific capacitance for one electrode (C_{sp}) was calculated according to the equation $C_{sp} = 4*C/m$,[4] where *C* is the capacitance of the cell.

The electrochemical characterization based on three-electrode electrolytic cell system used a Dropsens uStat400 potentiostat equipped with an Ag-AgCl electrode as the reference electrode, a platinum electrode as the counter electrode, and freestanding paper-like electrodes as the working electrode. The three electrodes were immersed in (1M) H_2SO_4 electrolytes at room temperature. CV was carried out in a potential range of 0 to 1 V at voltage scan rates of 10 mV/s. In this case, the specific capacitance of the working electrode is measured directly. For both electrochemical cell configurations, CV curves plots instant specific capacitance C_i (F/g) vs potential E (V), $C_i = I / (\Theta*m)$, where *I* is the instant electrical current, Θ is the voltage scan rate and *m* is the total mass of the active electrodes. The capacitance of the cell is calculate based on the equation $C = \int C_i\, dE / (2*(E_2-E_1))$, where E_2 and E_1 correspond to the cutoff voltages of the experiment.

S2. Transmission Electron Microscopy (TEM)

TEM images for GOCNT at an oCNT concentration of 10 wt. %, and for GOCNT sheets with oCNT concentrations above the agglomeration threshold are shown in Fig. S.2.

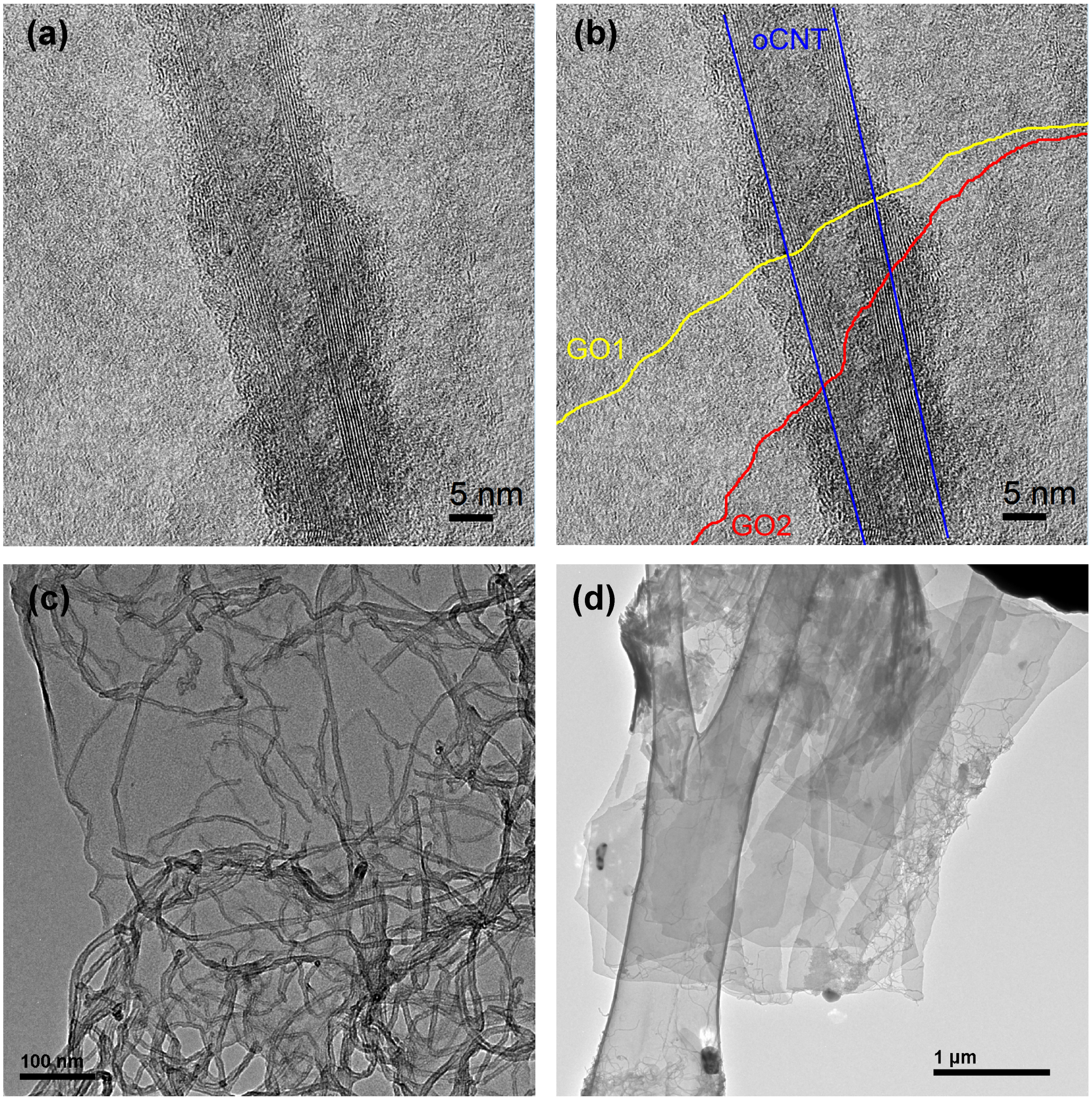

Fig. S2 (a) HRTEM image of GO-CNT10 showing a multi-walled oCNT embedded between two GO flakes. (b) The same image as in (a), but indicating in yellow and red color borderlines of GO flakes, and in blue color of CNTs. (c) TEM image of GO-CNT50 shows a high density of oCNTs with tendency to agglomerate and entangle on most GO flakes while (d) some sheets of GO-CNT50 are not covered at all by oCNTs (right image).

S3. Scanning electron microscopy (SEM)

A photograph (Fig. S3a) shows the typical macroscopic appearance of the assembled GO-CNT papers and may as well provide hints on their flexibility. The size of the papers is about 4 cm in diameter.

A FESEM micrograph (Fig. S3b) shows the external surface of GO-CNT50. It clearly reveals the presence of oCNTs interconnecting GO flakes. A SEM image at low magnification (Fig. S3c) provides information of the thickness of the GO-CNT papers, which typically amounts to about 30 μm. At higher magnification (Fig. S3d) the cross-section clearly shows the presence of uniformly self-assembled oCNTs on the GO flakes. SEM images of both, surface and cross-section thus reveal the formation of a uniformly nanostructured GO-CNT hybrid material.

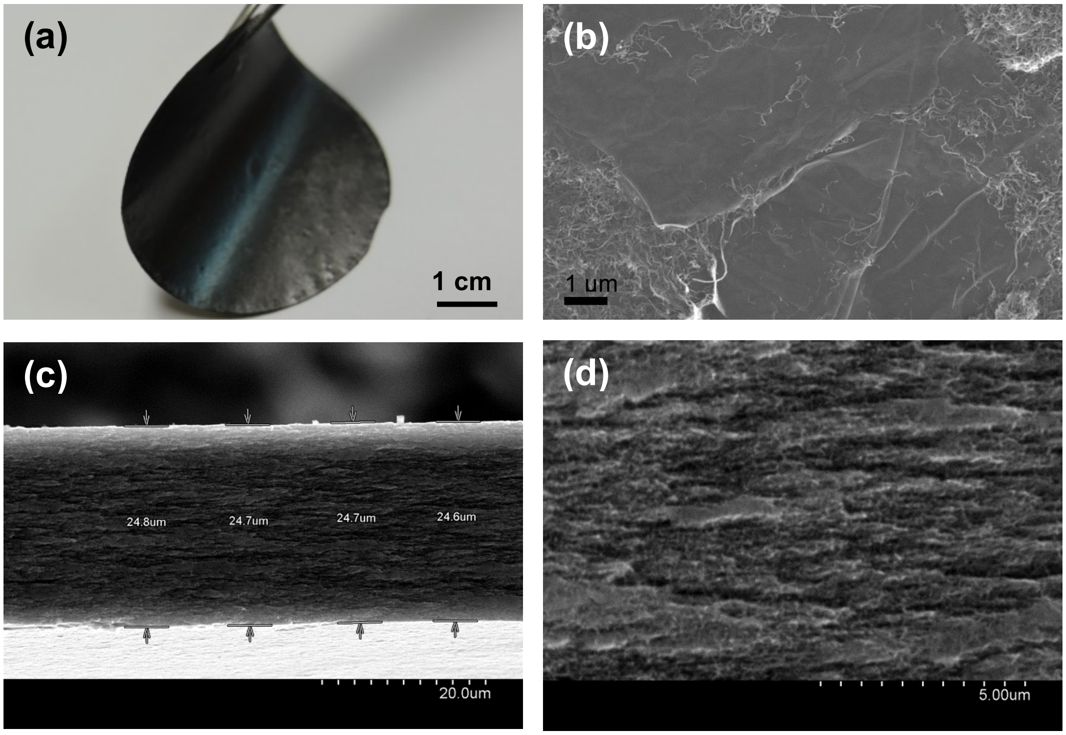

Fig. S3 Photograph of GO-CNT50 showing the macroscopic appearance of the paper (a), as well as the microscopic morphology of the external surface and cross-section, FESEM (b), and SEM (c, d) respectively. GO flakes and oCNT form a uniform nanostructured hybrid material while some oCNTs interconnect GO flakes with each other.

S4. Electrical transport measurements

Conductivity values are listed in Tab. S4 and depicted as a function of oCNT concentration in Fig. S4.

Tab. S4 Values for electrical conductivity for GOCNT papers with different loading of oCNT, GO-CNTx (x = 1, 5, 10, 15, 20, 50 wt. %), GO, and RGO.

GO samples	oCNT (wt. %)	Electrical conductivity (S/m)
**oCNT**	100	$2.2 \cdot 10^{3}$
**GO-CNT50**	50	$2.4 \cdot 10^{3}$
**GO-CNT20**	20	$7.3 \cdot 10^{2}$
**GO-CNT15**	15	$3.7 \cdot 10^{1}$
**GO-CNT10**	10	$2.4 \cdot 10^{1}$
**GO-CNT5**	5	5.3
**GO-CNT1**	1	4.6
**rGO**	0	5.0
**GO**	0	$2.4 \cdot 10^{-1}$

Improvements in conductivity through the incorporation of oCNTs can be classified by orders of magnitude: (i) order of 10^{-1} S/m for GO. (ii) order of 10^{0} S/m (i.e. improvement by one order of magnitude) for GO-CNT1 and GO-CNT5, as well as for rGO (GO thermally treated at 220ºC) thermally reduced rGO. This indicates that the incorporation of a small amount of oCNTs already allows for improvements, which otherwise are only achieved by thermal treatments; (iii) order of 10^{1} S/m for GO-CNT10 and GO-CNT15 (i.e. improvement by 2 orders of magnitude), indicating that incorporation of additional amounts of CNTs leads to further enhancement of conductivity; (iv) order of 10^{3} S/m for GO-CNT20, GO-CNT50 and oCNT, indicating that conductivity is fully determined by the presence of oCNTs, i.e. an entangled network of oCNTs. Please note that the electrical conductivity of oCNTs is significantly

higher than the one of GO and rGO. While GO with a value of 0.24 S/m itself is a mixed proton-electron conductor (the dominant transport mechanism depends on the relative humidity and the amount of intercalated water),[5] rGO (GO heat treated at 220 ºC, i.e. partially reduced with remaining OFGs) with a value of 5 S/m shows low electron conduction most likely dominated by variable-range hopping related to the presence of remaining OFGs.[5] Conductivity values for rGO comparable to (or even higher as) oCNTs require annealing treatments at temperatures of about 700 ºC.[1] Therefore, the conductivity behaviour of our hybrid (r)GO-CNTx papers, being parent or being partially reduced at 220 ºC is dominated by the presence of oCNTs providing a percolating electron conducting network. A percolation-like behaviour with a change of conductivity regime at about 10 to 15 wt. % of oCNTs is observed for GO-CNTx papers and shown in Figure 1d of the main manuscript.

S5. TWO DIMENSIONAL X-RAY SCATTERING (2D XRD)

The paper assemblies are studied using two-dimensional X-ray diffraction (2D XRD) patterns (Fig. S5a,d), taken in transmission for samples GO, rGO, GO-CNT and rGO-CNT. Vacuum conditions increase the signal to noise ratio and allow for the observation of a weak isotropic diffuse scattering signal. Diffractograms for GO and RGO acquired under ambient and vacuum conditions, in the direction indicated by the red line (Fig. S5a, d). are reported in (Fig. S5b, e).

They provide information about the interlayer stacking. The diffractogram of GO taken under ambient conditions reveals a strong 001 peak located at a GO interlayer sheet distance of 7.2 Å, comprising the presence of OFGs and confined water molecules between the sheets**.** In agreement with other works, this peak is denoted as "hydrophilic peak".[6] Under vacuum conditions this peak shifts to a somewhat lower interlayer distance of 6.9 Å, thus indicating the (partial) removal of (physisorbed) water molecules confined in between the layers. An additional small intensity 001 peak can be seen at an interlayer distance of about 3.4 Å on the GO diffractogram. It is of graphitic character and thus may be denoted as "hydrophobic" peak. Its appearance indicates the presence of stacked graphitic regions neither containing water nor OFGs. The average number of stacked layers for the hydrophobic and the hydrophilic peak, as calculated from the peak width (FWHM), amounts to 3-4. Importantly, for rGO (i.e. GO treated at 220ºC) no "hydrophilic" peak is seen anymore neither under air nor under vacuum conditions. Instead, the "hydrophobic" peak now presents an asymmetric shape indicating a distribution of interlayer distances towards values somewhat larger than 3.4 Å. It is attributed to the removal of water and OFGs. Apparently, this process is not occurring homogeneously and thus results in imperfect stacking. The angular distribution of the hydrophilic and hydrophobic peaks is anisotropic their intensity being maximum perpendicular to the film surface showing that the stacked GO sheets are preferentially oriented parallel to the films surfaces.

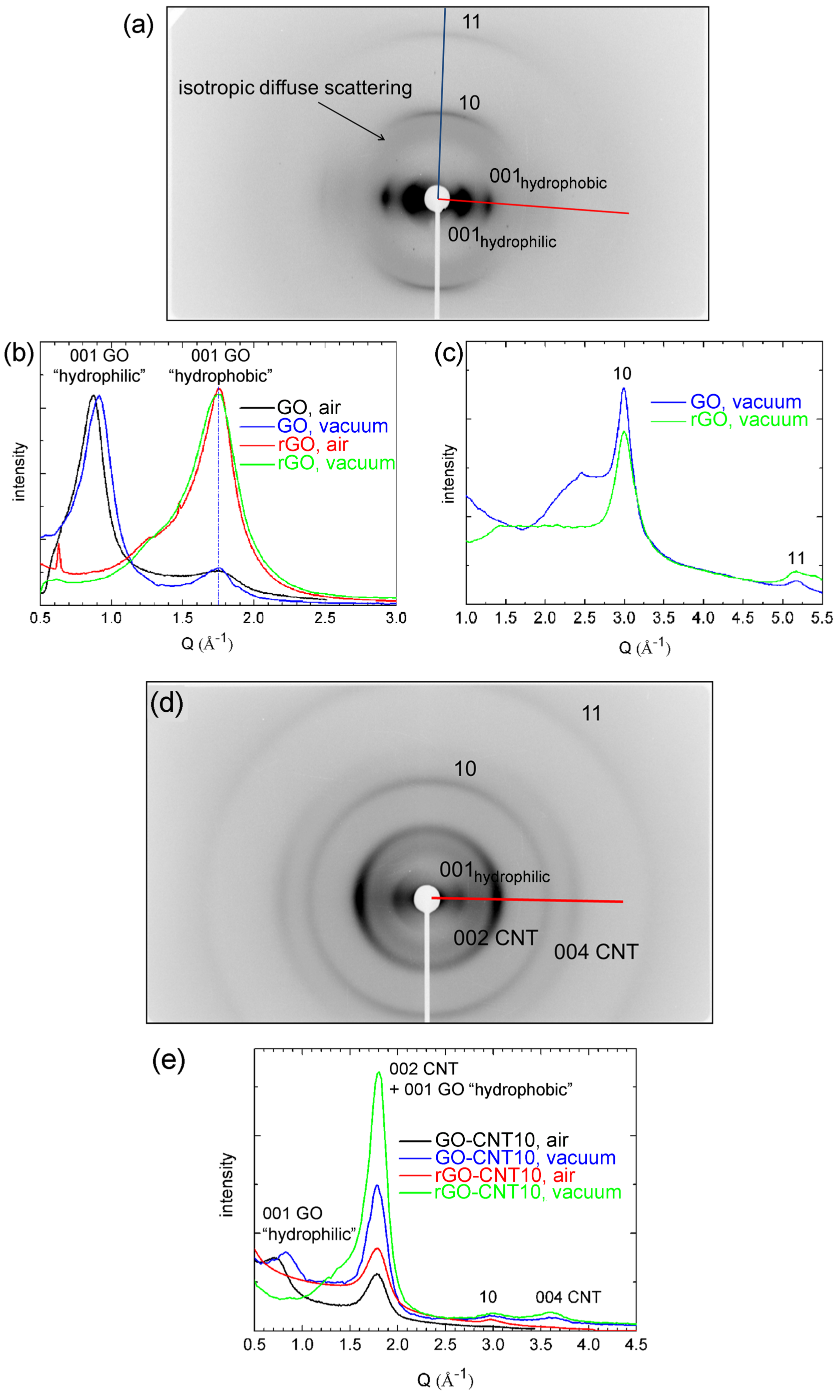

Fig. S5 (a) 2D XRD pattern of GO paper under vacuum. (b) X-ray diffractogram along the red line for GO, and rGO papers in air and vacuum conditions. (c) X-ray diffractogram along the blue line for GO, and RGO in air and vacuum. (d) 2D XRD pattern of GO-CNT10 paper under vacuum. (e) X-ray diffractogram along the red line for GO, and rGO papers in air and vacuum conditions. Scattered peaks are indexed $001_{hydrophilic}$, $001_{hydrophobic}$, 002 and 004 CNT, 10, and 11.

The diffractograms along the direction indicated by the blue line in Fig. S5b provides information about the in-plane arrangement of carbon atoms for GO and rGO (Fig. S5d). Both samples show the typical 10 and 11 diffraction peaks corresponding to a lattice parameter value of d_{10} = 2.46 Å and a C-C bond length of 1.42 Å. Moreover, for GO a broad diffuse scattering signal is observed for Q wavevectors ranging from 2 to 2.7 $Å^{-1}$. It is of isotropic character and most likely refers to the presence of randomly orientated water molecules in close contact to the OFGs of the GO sheets. This broad diffuse scattering signal completely disappears for the rGO paper (GO paper thermally treated at 220 ºC).

In the case of GO-CNT10, a clear predominance of the hydrophobic 001 peak over the hydrophilic 001 peak can be seen (Fig. S5e). The angular pattern for GOCNT10 is shown in Fig. S5d. Here, due to the presence of the multiwall oCNTs randomly distributed in between the GO sheets, the strong 002 interlayer scattering of the oCNTs[7] has to be taken into account, which now overlaps with the hydrophobic 001 GO contribution. CNTs are found to have their long axis preferentially oriented within the film plane, with their 002 ring having maximum intensity perpendicularly to the film surfaces. The low intensity of the "hydrophilic" 001 peak indicates that the amount of the stacked hydrophilic GO sheets containing intercalated water is relatively weak. As for the case of GO, the 001 peak of GO-CNT10 shifts towards lower interlayer distances under vacuum, i.e from 8.85 to 7.6 Å, indicating (partial) removal of intercalated physisorbed water. After the 220 ºC treatment, once again, the original hydrophilic peak of GO is not longer observed in rGO-CNT10 and subsequent vacuum conditions do not affect the diffractogram.

S6. Thermogravimetric analysis (TGA)

Thermogravimetric analyses (TGA) for GO-CNT papers with oCNT concentrations of 15, 20, 50, and 100 wt. % are shown in Fig. S6a,b. It clearly can be noticed that beyond a concentration of 15 wt. % an important additional weight loss region appears between 260 to 400 ºC. As can be seen from the TGA curve of the pure oCNT paper sample, the corresponding weight-losses, which are rather broad, are characteristic for the decomposition of oCNTs themselves, including both their functional oxygen groups and the carbon backbone itself. This suggests that for concentrations higher than 15 wt. % the behaviour of GO-CNT papers is increasingly governed by oCNTs, most likely present in the form of larger agglomerates forming entangled networks, in agreement with observations from conductivity measurements (see section S.4). Furthermore, the TGA trends for temperature higher than 400 ºC, as well as the final TGA values reached at 800 ºC for samples with concentrations higher than 15 wt. %, approach the trend for oCNTs (Fig. S6a). Lower values for GO-CNT20 and GO-CNT may suggest the formation of inhomogeneous samples, composed of multilayer stacks of GO sheets and dense oCNT networks, in contrast to rather homogeneous GO-CNT stacks for oCNT concentrations below 15 wt. %, in agreement with the HRTEM findings (see section S2).

TGA curves for GO and RGO papers are depicted in Fig. S6c,d. The overall TGA mass losses at 800 ºC for pristine GO and rGO (GO heated at 220 ºC) amount to 53 wt. % and 34 wt. %, respectively. In other words, GO shows a lower thermal stability with respect to rGO at 800 ºC. Two important regions for the mass losses are observed: The region up to 130 ºC, indicated by PW, refers to losses related to the removal of physisorbed water. The region from 130 to 260 ºC, indicated by CW, denotes the removal of chemisorbed water and OFGs, as outlined in the article. The “water threshold” is located at about 260 ºC, once having passed the two temperature zones. Interestingly, for all papers measured, GO reveals the highest mass losses after having passed the regions from room temperature to 130 ºC and 130 ºC to 260 ºC, namely 15 wt. % and wt. 22 wt. %, respectively, as presented in Fig. S6e. By contrast, rGO

accounts for mass losses of 5 wt. % (130 ºC) and only 3 wt. % (260 ºC). The improved thermal stability at 260 ºC for rGO paper (GO heated at 220 ºC) is explained by the irreversible desorption of CW and OFGs after heating the paper at 220 ºC. Accordingly, the temperature at 260 ºC is marked as "water threshold" in Fig. S6c,d. The mass loss calculations for this temperature range are illustrated in Fig. S6e. The clear trend for the decrease of water with increased oCNT content after having passed the water threshold goes along with the increment of electrical conductivity for GO-CNT samples and the ranges of conductivity established (see section S4). Similarly, the TGA data also indicate a threshold at about 15 wt. %.

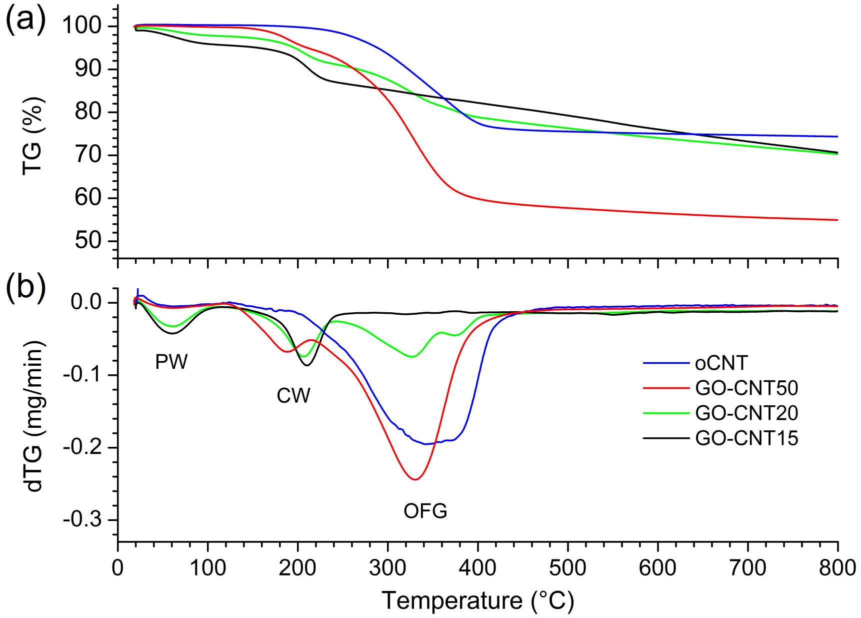

Fig. S6. Thermogravimetric analyses. (a, b) Relative mass loss (TGA) and differential analysis (DTG) of paper samples of GO-CNT15, GO-CNT20, GOCNT50, oCNTs.

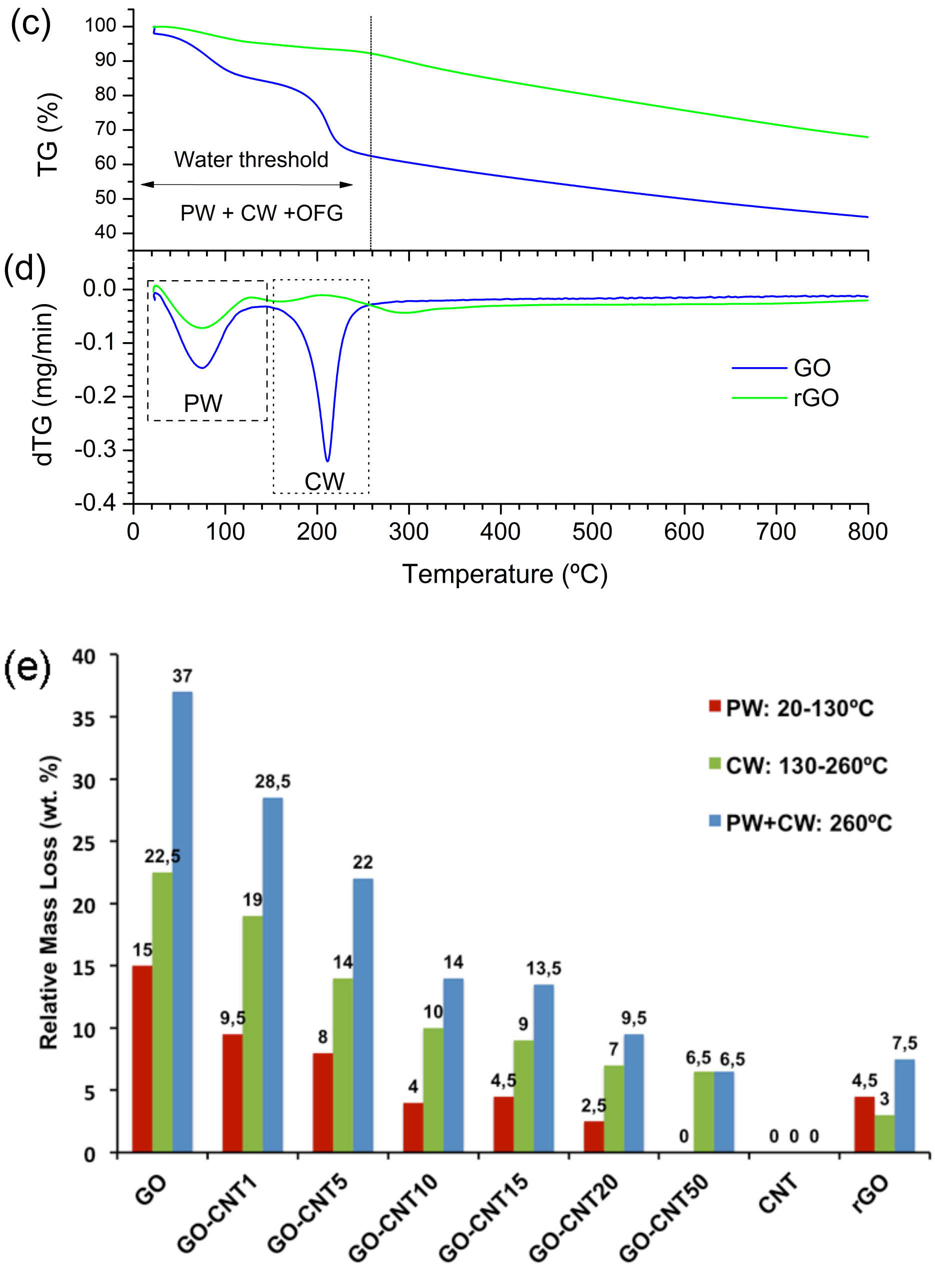

Fig. S6. Thermogravimetric analyses. (c, d) Relative mass loss (TGA) and differential analysis (dTG) of pristine GO and 220 ºC heated paper rGO. (e) Relative mass loss values (wt. %) derived from TGA of GO below the water threshold temperature at 260 ºC. The mass loss calculations are divided in sections PW (20 - 130 ºC) and CW (130 - 260 ºC), which are related to PW and CW removal, respectively. PW+CW denotes the sum of the weight losses at 260 ºC. The obtained values indicate the thermal stability after having passed the water threshold at 260 ºC. Lower values denote higher thermal stabilities.

S7. X-ray photoelectron spectroscopy (XPS)

XPS spectra in Fig. S7 show the deconvoluted C1s and O1s core shell spectra of GO, CO-CNT10, rGO and rGO-CNT10 papers. The spectra are discussed in the main article. Most important to highlight is the significant decrease of the contribution of (chemisorbed) water in GO-CNT10, as well as in rGO and rGO-CNT10 as seen from the O1s spectra. The deconvoluted C1s and O1s core shell spectra for oCNTs with respect to GO and rGO are shown for comparison.

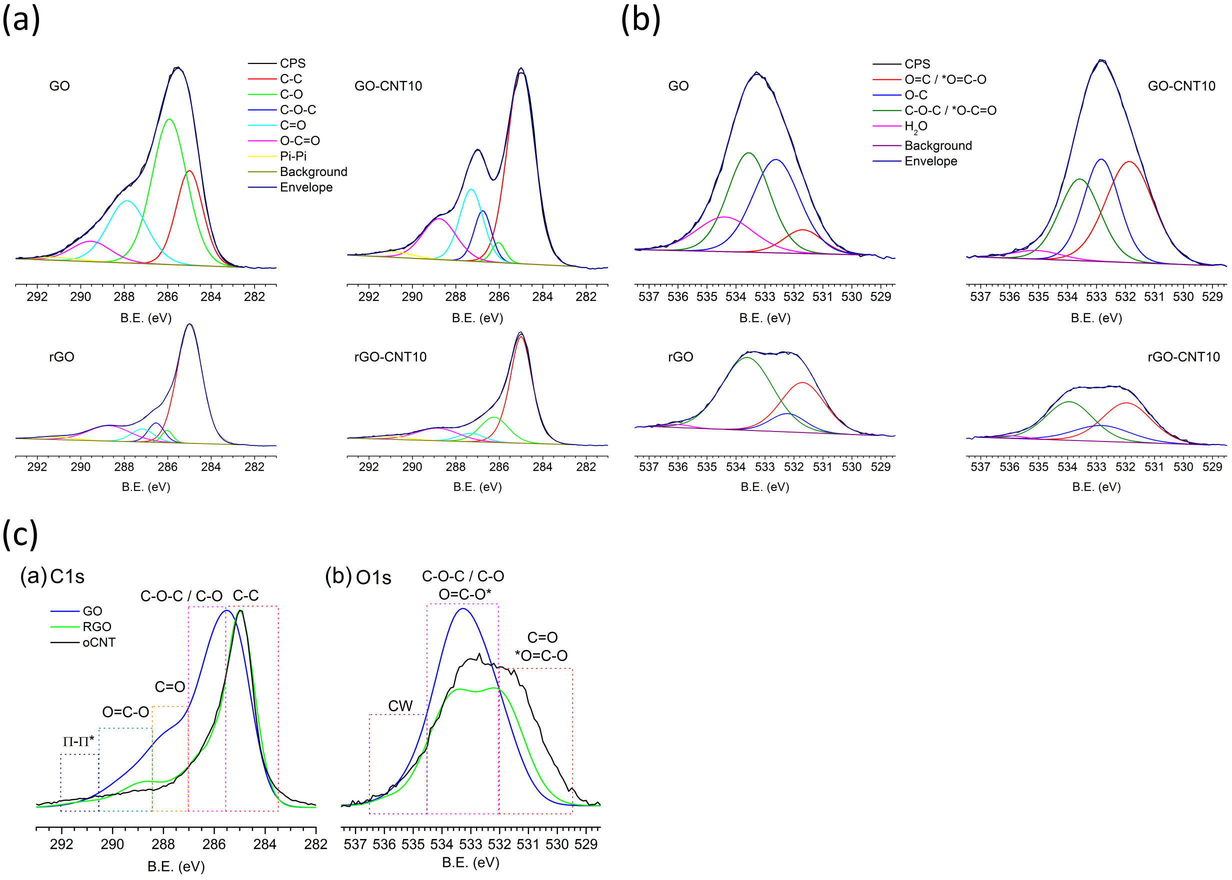

Fig. S7. Deconvoluted core shell spectra of the (a) C1s core shell spectra and (b) O1s core shell spectra for GO, GO-CNT10, rGO and rGO-CNT10. (c) C1s and O1s spectra for oCNT, GO and rGO. Shown are the contributions of the indicated functional groups, the background and the overall fitted envelope.

S8. Fourier Transform Infrared Spectroscopy (FTIR)

FTIR spectra for oCNTs are jointly shown with the ones for GO and rGO papers in Fig. S8**.** A clearly distinct spectrum is observed for oCNT paper. In contrast to GO and rGO, the OH stretch band (left panel) is rather narrow and only ranges down to 3000 cm^{-1}. Apparently, no intermolecular hydrogen bonds are formed in oCNT, which would contribute to anharmonic broadening of the OH stretch band below 3000 cm^{-1}, as explained in the article. The right panel shows the region from 1900 cm^{-1} down to 500 cm^{-1}. The spectra for oCNT in this zone shows the characteristic modes related to carboxylic and carbonyl groups (overall C=O and C-O stretch bands), i.e. the typical types of OFGs attached to oCNTs. Due to their low amount in comparison with GO, their intensities are lower as compared to GO. Their positions also are slightly shifted due to considerably less amount of water interacting with the C=O and C-O stretching modes. The intensity for the water scissor mode (1626 cm^{-1}) and the OH in-plane bending modes at 1400 cm^{-1} and 1385 cm^{-1} are indicative for the presence of relatively low amounts of water in oCNT.

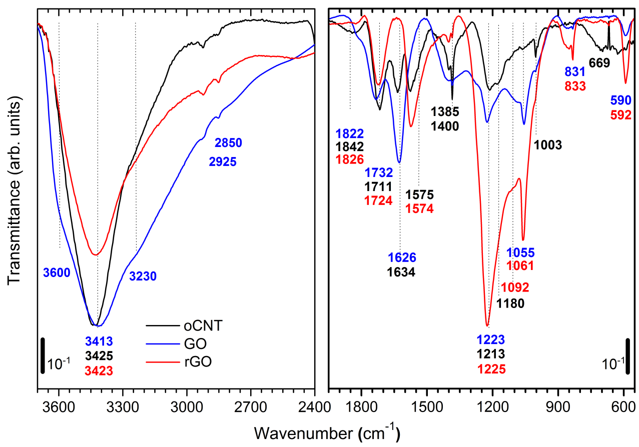

Fig. S8. FTIR spectra for oCNT, GO, and rGO paper. Left panel shows the region from 3600 to 2400 cm^{-1}, typical for OH stretch modes. Right panel shows the region from 1900 to 500 cm-1, corresponding to the “fingerprint” region characteristic for the OFGs attached to oCNTs and GO papers.

S9. Cyclic Voltammetry (CV)

The practical implications of the removal of CW in GO-CNT papers were studied in electrochemical applications and the evolution of the capacitance derived from cyclovoltammetric measurements. Fig. S9a shows the electrochemical performance of GO-CNT50 and GO-CNT20 papers, before and after heating at 220 ºC, probed by cyclic voltammetry (CV) in a two-electrode configuration.

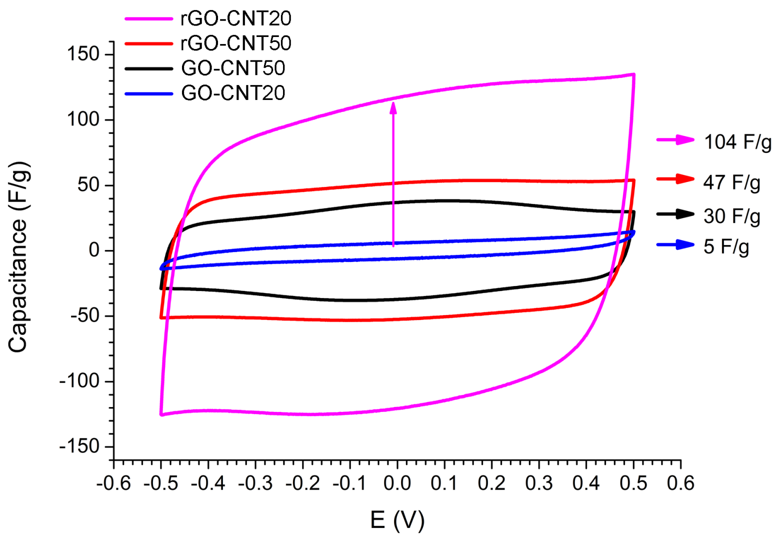

Fig. S9.a CV curves of GO-CNT50 and GO-CNT20 obtained before and after heating both samples at 220º C at a fixed scan rate of 50 mV/s. The specific capacitance (F/g) indicated for each curve is calculated for two-electrode electrolytic cell configuration in (1M) Na_2SO_4.

GO-CNT50 reveals higher specific capacitance (30 F/g) than GO-CNT20 (5 F/g). GOCNT50 shows a nearly rectangular behaviour and maximum anodic and cathodic specific capacitance peaks at 0.11 V and -0.11 V, respectively, which indicate the mixed contribution of electric double layer capacitance (EDLC) and pseudocapacitance (PSC) processes. Conversely, the specific capacitance of GO-CNT20 linearly increases

slightly towards the edges of the voltage window (-0.5 V, 0.5 V), which is characteristic of more resistive electrochemical electrodes.

In order to explain these results, we should consider the effect of the content of oCNT on the GO-CNT assembly structure. The higher the content of oCNTs the higher is the probability of its agglomeration and entanglement. In the case of GO-CNT50, agglomeration is the dominant factor (Fig. S2), thus oCNTs agglomerate within GO sheets leading to a nanoporous structure with higher electrochemical surface contribution of oCNT with respect to GO-CNT20. Consequently, the permeation of the electrolyte throughout the internal surface area of the tubes should be easier for GO-CNT50 than for GO-CNT20. Nevertheless, as the loading of CNT increases the electrochemical performance of GO-CNT should resemble the rectangular electric double layer capacitance behavior for pure oCNT paper electrodes where the contribution of the internal surface of GO remain almost blocked by the high content of oCNT, which are interacting among themselves and with the OFG of the GO via H-bonds, as discussed in the article.

On the contrary, as the loading of CNT decreases the contribution of PSC, most likely attributed to the effect of ion intercalation and protonation-deprotonation reactions, should increase due to the relative higher density of electrochemically redox OFGs of GO. However, the resistive performance of GO-CNT20 seems to undermine the accessibility of the electrolyte towards the specific surface area of GO. In this case, despite the more efficient insertion of oCNTs in between the GO's nano-channels the resulting porous structure is not yet un-blocked for an easy permeation of the electrolyte. Therefore, the electrochemical performance of GO-CNT20 is reduced, mostly due to the poor wettability of the electrolyte.

After the thermal treatment, the electrochemical performances of the GO-CNT hybrids clearly reverse the situation encountered before. The CV of rGO-CNT20 now reveals a significantly enhanced rectangular shape with PSC contribution that improves its specific capacitance up to 100 F/g, thus

doubling the capacitance of rGO-CNT50. These results can be explained considering that CW can hamper the electrolyte permeation into the nano-channels of GO. CW might be the origin of diffusion limitations for GO-based electrodes in aqueous electrolytes not only blocking the permeation of the electrolyte but also, and even more importantly, deactivating OFGs as electrochemical redox centres for improved PSC. Therefore, the electrochemical area of GO might be not efficiently exploited without the removal of CW. As discussed, thermal treatments above 200 ºC induce the removal of CW. This eases the permeation of the electrolyte towards the accessible OFGs, which become "activated".

However, as CW and OFGs are removed, the interlayer distance of GO decreases and narrows the average size of the nano-channels of the electrode, which in turn might hindering the permeation of the electrolyte. Another counter-effect of the removal of CW is the structural degradation of GO by the steam reaction. In the case of GO-CNT hybrid papers, the insertion of oCNTs in between the GO sheets avoids the shrinkage effect of the nano-channels and increases the porosity of the electrode favouring the permeation of the electrolyte upon removal of CW. Furthermore, the interaction via H-bond of the GO-oCNT units reduces the amount of CW to be removed and preserves most OFG from being decomposed after the 220 ºC thermal treatment. As result, both EDLC and PSC contribution of oCNTs and GO can be simultaneously enhanced. This explains why both rGOCNT50 and rGOCNT20 improve their specific electrochemical capacitance.

Nonetheless, the efficient insertion of oCNT instead of the agglomeration of oCNTs should prevails to obtain the maximum potential contribution of PSC from GO. Otherwise, the GO units shrink more easily upon removal of CW. For instance, the higher capacitance of rGO-CNT20 with respect to rGO-CNT50 is due to the more efficient insertion of oCNTs of the first compared with the agglomeration tendency of the second.

In order to evaluate the GO-CNT assembly as energy storage materials, we also have studied the electrochemical performance of a single electrode in a three-electrode electrolytic cell configuration.

Our results indicate that an optimum insertion of oCNTs at about 10 to 15 % of oCNTs increases the PSC contribution of GO achieving the highest specific capacitance for rGO-CNT15 with values up to 156 F/g, which denotes an improvement by 75% with respect to the capacitance of rGO papers (Fig. S9b and Tab. S9a).

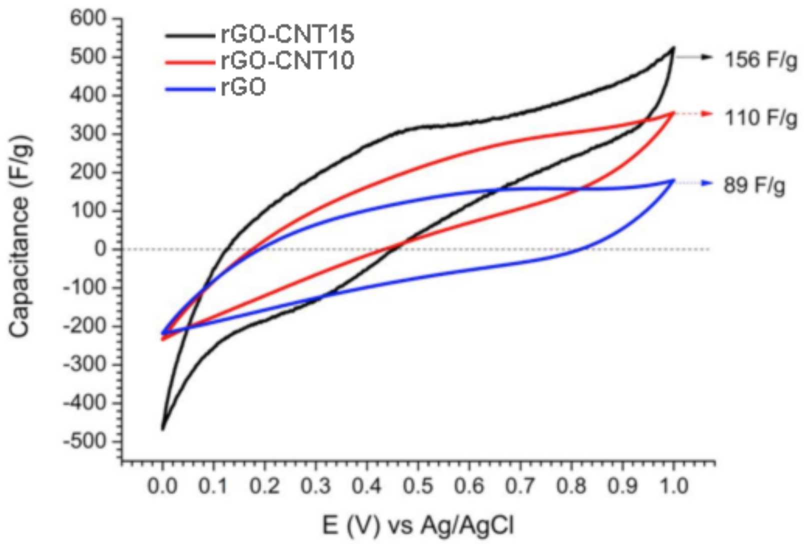

Fig. S9b CV curves of the 220 ºC heated samples rGO-CNT15, rGO-CNT10, and rGO in (1M) H_2SO_4 at 10 mV/s (a). The specific capacitance (F/g) indicated for each curve is calculated considering the anodic contribution for three-electrode electrolytic cell configuration.

Tab. S9a Specific capacitance of rGO-CNTx papers (x = 50, 20, 15, 10, wt. %), oCNT and rGO. Improvement of specific capacitance with respect to rGO is indicated in last column.

GO-CNTx paper	Specific Capacitance (F/g)	Improvement (%)
oCNT	15	
rGO-CNT50 (GO-CNT50)	47 (30)	
rGO-CNT20 (GO-CNT20)	104 (5)	17
rGO-CNT15	156	75
rGO-CNT10	110	23
rGO	89	

Tab. S9b Selected literature values for specific capacitance of rGO-CNT papers showing their maximum performance for a CNT concentration of about 10 wt. %.

Reference	Sample	Specific Capacitance (F/g)	Improvement (%)	Comment
8	rGO	116		3 µm thick paper.
	rGO-CNT12.5	156	34	Prep.: Mix of GO water disp and CNT powder.
9	rGO	248		240 nm thick films on Ti.
	rGO-CNT12.5	279	12.5	Prep.: Mix of GO water disp. and CNT water disp.
10	rGO	190		Hydrothermal treated films.
	rGO-CNT10	181 (224)	-5 (18)	Prep.: Mix of GO water disp. and SDBS stabilized CNT disp + PTFE binder. Value in parenthesis refers to graphene contribution assuming a parallel capacitance contribution
11	rGO	75, 50 (130, 100)		Thin film on Ti;
	rGO-CNT9	125, 113 (240, 237)	66,126 (85, 137)	Prep.: GO ethanol disp + CNT powder. (values in parenthesis refer to negative potential window)

Although absolute values of the specific capacitance are not really comparable with those from literature (owing to a wide spread in the conditions for sample preparation, film/paper processing, sample characteristics (including thicknesses), as well as the electrochemical parameters), it seems that maximum improvements are achieved in a narrow CNT concentration range of 10-15 wt. % (see Tab. S8b). This is based on effectively enhanced pore-size and increased conductivity provided by (homogeneously) intercalated CNTs, as typically pointed out in literature.[8-10] However, it is important to

note, that in our case the considerable improvement of capacitance by 75 % is achieved by using oxidized carbon nanotubes (oCNTs). Therefore, beyond the effects of enhanced porosity and conductivity provided by carbon nanotubes, the influence of the removal of chemisorbed water due to the presence of oCNTs (i.e. the interaction of oCNTs with OFGs of GO achieved in the in-situ liquid phase processing steps) additionally has to be taken into account. It becomes clear that the removal of chemisorbed water provides additional access of the electrolyte to the active surface sites. This is further underlined by the significant enhancement of the specific capacitance achieved through thermal reduction at 220 ºC of the hybrid papers (see Tab. 9a). It not only explains the highest capacitance values achieved, but even more, the electrochemical behaviour as a function of the oCNT concentration. Therefore, the removal of chemisorbed water by oCNTs plays an important role for tailoring the electrochemical performance.

Summarizing, the efficient (homogeneous) insertion of oCNTs in between the GO sheets is key to improve the specific capacitance of GO-CNT electrodes by playing two different roles: (a) They cause the removal of most of CW before thermal treatments, thus reducing the degradation effects of the steam reactions on the carbon structure while maintaining the density of OFGs on the GO sheets. This favours the enhancement of EDLC and PSC contributions to the overall capacity; (b) they contribute to the development of enhanced pore sizes in GO and avoid its shrinking or collapsing after subsequent thermal treatments thus favouring the ion-accessibility to the electrochemically active sites of rGO.

REFERENCES

1 C. Vallés, J. David Nuñeez, A. M. Benito and W. K. Maser, *Carbon*, 2012, **50**, 835-844.

2 D. K. Schroder, *Semiconductor material and device characterization*, John Wiley & Sons, Inc., Hoboken, New Jersey (USA), 3rd edn., 2006.

3 B. E. Warren, *X-ray diffraction*, Addison-Wesley Pub. Co., Reading, Mass., 1969.

4 M. D. Stoller and R. S. Ruoff, *Energy Environ. Sci.*, 2010, **3**, 1294.

5 T. Bayer, S. R. Bishop, N. H. Perry, K. Sasaki and S. M. Lyth, *ACS Appl. Mater. Interfaces*, 2016, **8**, 11466-11475.

6 J. Zhu, C. M. Andres, J. Xu, A. Ramamoorthy, T. Tsotsis and N. A. Kotov, *ACS Nano*, 2012, **6**, 8357-8365.

7 P. Landois, M. Pinault, S. Rouzière, D. Porterat, C. Mocuta, E. Elkaim, M. Mayne-L'Hermite and P. Launois, *Carbon*, 2015, **87**, 246-256.

8 Z.-D. Huang, B. Zhang, R. Liang, Q.-B. Zheng, S. W. Oh, X.-Y. Lin, N. Yousefi and J.-K. Kim, *Carbon*, 2012, **50**, 4239-4251.

9 Z.-D. Huang, B. Zhang, S.-W. Oh, Q.-B. Zheng, X.-Y. Lin, N. Yousefi and J.-K. Kim, *J. Mater. Chem.*, 2012, **22**, 3591-3599.

10 Y. Wang, Y. Wu, Y. Huang, F. Zhang, X. Yang, Y. Ma and Y. Chen, *J. Phys. Chem. C*, 2011, **115**, 23192-23197.

11 X. Cui, R. Lv, R. U. R. Sagar, C. Liu and Z. Zhang, *Electrochim. Acta*, 2015, **169**, 342-350.
